# Structure Based Virtual Screening Studies to Identify Novel Potential Compounds for GPR142 and Their Relative Dynamic Analysis for Study of Type 2 Diabetes

**DOI:** 10.3389/fchem.2018.00023

**Published:** 2018-02-14

**Authors:** Aman C. Kaushik, Sanjay Kumar, Dong Q. Wei, Shakti Sahi

**Affiliations:** ^1^State Key Laboratory of Microbial Metabolism and School of Life Sciences and Biotechnology, Shanghai Jiao Tong University, Shanghai, China; ^2^School of Biotechnology, Gautam Buddha University, Greater Noida, India; ^3^Molecular Structural Biology Division, CSIR-Central Drug Research Institute Lucknow, Lucknow, India

**Keywords:** GPR142, virtual screening, pharmacophore hypothesis, VSW, IFD, systems biology, MD simulation, type 2 diabetes mellitus (T2DM)

## Abstract

GPR142 (G protein receptor 142) is a novel orphan GPCR (G protein coupled receptor) belonging to “Class A” of GPCR family and expressed in β cells of pancreas. In this study, we reported the structure based virtual screening to identify the hit compounds which can be developed as leads for potential agonists. The results were validated through induced fit docking, pharmacophore modeling, and system biology approaches. Since, there is no solved crystal structure of GPR142, we attempted to predict the 3D structure followed by validation and then identification of active site using threading and *ab initio* methods. Also, structure based virtual screening was performed against a total of 1171519 compounds from different libraries and only top 20 best hit compounds were screened and analyzed. Moreover, the biochemical pathway of GPR142 complex with screened compound2 was also designed and compared with experimental data. Interestingly, compound2 showed an increase in insulin production via Gq mediated signaling pathway suggesting the possible role of novel GPR142 agonists in therapy against type 2 diabetes.

## Introduction

Worldwide around 382 million people have been diagnosed with type 2 diabetes mellitus. With an increasing incidence of type 2 diabetes, this disease has engrossed great concern (Du et al., 2012). It is characterized by high level of blood glucose resulting from synergistic effect of reduced insulin production and insulin resistance by the pancreatic β-cell (Lizarzaburu et al., 2012). One of the important features is the deterioration of glucose control progressively over a period of time. The hyperglycemia increases the risk of cardiovascular complications in patients with diabetes that includes stroke, neuropathy, nephropathy, and retinopathy. Hence, to prevent chronic diabetic complications, it is important to have effective glycemic control (Ahrén, 2009). Presently, sulfonylureas and meglitinide as insulin secretagogues are being used for treatment of type 2 diabetes in patients (Winzell and Ahrén, 2007). However, these compounds lead to insulin release independent of blood sugar level and result into hypoglycemia. The novel glucose stimulated insulin secretagogues such as 5 GLP-1 analogs, DPP-IV inhibitors, GPR119 agonists and GPR40 agonists have opened new and alternative treatment against Type 2 diabetes (Augeri et al., 2005). Also, diabetes requires multi-drug therapies with a new intervention every few years to have better control. Hence, it is essential to develop the therapies that can lower the glucose level in the blood without risk of hypoglycemia.

GPR142, an orphan G protein-coupled receptor, is predominantly expressed in pancreatic β-cells (Overton et al., 2008). The stimulation of GPR142 by tryptophan initiated intracellular signal transduction leads to enhanced glucose dependent insulin secretion in isolated mouse islets (Kahn et al., 2006). Hence, GPR142 can be a potentially advantageous drug target for diabetes therapy and can provide an alternative therapy with reduced risk of hypoglycemia. However, the 3D-structure and signaling pathways downstream of GPR142, and its mechanisms are poorly characterized.

In this study, we have reported the structure based virtual screening to find the hit compounds that can be used to develop potential leads for novel agonists of GPR142 (Gund et al., 1974; Hopfinger, 1985; Guner, 2000). The compounds were validated through induced fit docking studies whilst ligand based virtual screening was employed for pharmacophore modeling to derive the structural requirements crucial for receptor binding (Dixon et al., 2006b). A complete network pathway was constructed, and kinetic studies were carried out for the screened compounds binding to GPR142 to better understand the mechanism as well as effect on insulin secretion.

## Methodology

### 3D structure prediction and validation

**Figure 1:**
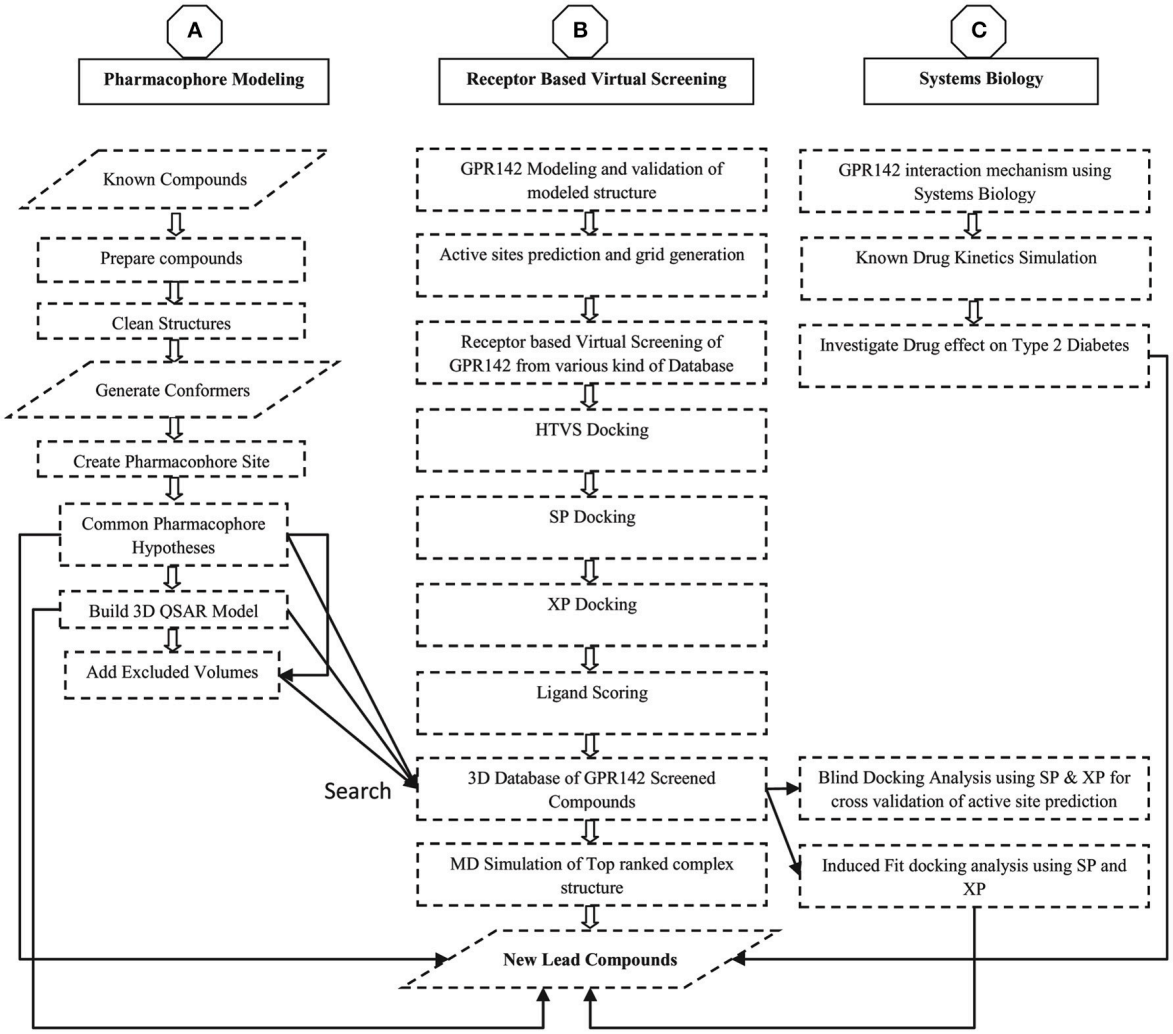
Flow chart diagram for the modeling, Virtual screening, Pharmacophore generation, and 3D database screening for potential lead compounds as GPR142 agonists.

The sequence of GPR142 (UniProt ID: Q7Z601 and GenBank ID: NP-861455.1) was retrieved from UniProt Database (Chen et al., 2017; Pundir et al., 2017). As there is no solved crystal structure of GPR142 and sequence showed a homology of only 21%, 3D structure prediction was done using threading and *de-novo* methods. The threading approach was based on sequence-structure alignment that includes searching of homologous protein structures in the PDB (Lemer et al., 1995). Whereas, ab initio modeling was based on conformational search under the guidance of a designed energy function and model precision was highly defined by the protein sequence length i.e., <100 amino acid residues produced better results (Hardin et al., 2002). A Delta-type opioid receptor chimeric protein [PDB ID: 4N6H] (Fenalti et al., 2014) was initially selected as reference template for build secondary structure of GPR142 using Modeler v9.8 program (B. Webb, A. Sali. 2014, Eswar et al., 2008). The 3D modeled structure of GPR142 was then prepared using Protein Preparation Wizard (Sastry et al., 2013). The model was further validated by various modules available in SAVE server (Lüthy et al., 1992; Colovos and Yeates, 1993; Laskowski et al., 1993; Hooft et al., 1996; Pontius et al., 1996; Vaguine et al., 1999; Benkert et al., 2008, 2009a,b, 2011). The methodology adapted in this manuscript is shown as flowchart in Figure 1.

### 3D tunnel and domain prediction

To understand the mechanism of ligand movement and probable binding sites, tunnels were located in the modeled structure of GPR142 using CAVER2.1 (Beneš, 2008; Beneš et al., 2010). Domain predictions were performed using TMbase (Hofmann and Stoffel, 1993) and GPCRHMM server (Wistrand et al., 2006). TMbase and GPRCRHMM algorithm were based on statistical and HMM analysis, respectively.

### Active site prediction

Sitemap module of Schrödinger software suite (Halgren, 2007, 2009) was used to predict the binding site of GPR142. SiteMap used the interaction energies to locate the energetically favorable regions. It initially traced the sites that include a set of site points on a grid. The numbers of site points for a site were set to 15 and the number of sites to be found was set to 5. More restrictive definition of hydrophobicity and OPLS force field was used for all the calculations.

### Receptor based virtual screening

The structure based virtual screening analysis was performed using Virtual screening workflow of Schrödinger software suite (Friesner et al., 2004, 2006; Halgren et al., 2004) against different libraries of compounds. All the ligand structures present in the databases were in 2D SDF format and converted to 3D for docking studies. OPLS 2005 force field (Jorgensen and Tirado-Rives, 1988; Jorgensen et al., 1996; Shivakumar et al., 2010) was used for geometry optimization by truncated newton conjugate gradient (TNCG) minimization. Receptor grid was generated using centroid of active site residues with van der Waals scaling factor of 1.0 and partial charge cutoff at 0.25. LigPrep (Schrödinger Release, 2015) was used to prepare the ligands with Epik (Shelley et al., 2007; Greenwood et al., 2010) at 7 ± 2.0 pH units to expand protonation and tautomeric states with OPLS2005 force field. Low energy stereoisomers were generated for each ligand and ones holding low energy 3D structures with correct chiralities were retained. The different used libraries were namely; (1) Zinc, (2) SchrodingerDB, (3) TimTec, (4) PUBCHEM, (5) Not annotated NCI, (6) Marine, (7) DrugBank (Approved, Biotech, ILLICIT, Investigational, Nutraceutical, Withdrawn), (8) ChemBank, (9) Anti-HIV NCI, (10) DrugLikness NCI, (11) Asinex Ltd., and (12) ChEBI (Grotthuss et al., 2004; Irwin and Shoichet, 2005). Virtual screening was carried out in three phases: (a) Structure based virtual screening (HTVS), (b) Standard Precision (SP), and (c) (XP); Top 20 screened compounds were selected following virtual screening based on docking score range (−13.041 to −8.0).

### Validation

The top 20 compounds screened through virtual screening were validated through blind docking, induced fit docking, and pharmacophore generation. The validated compounds were then used for further studies.

#### Blind docking

Blind Docking using SP and XP was done without specifying the active site residues. The epik state penalties were added to the docking score (Shelley et al., 2007; Greenwood et al., 2010). Scaling of van der Waals radii was also set to 0.8 and partial charge cutoff at 0.15. The number of poses generated per ligand was 100.

#### Induced fit docking

The 20 screened compounds were evaluated by induced fit docking (IFD) (Farid et al., 2006; Sherman et al., 2006a,b) wherein flexibility was imparted to the residues in active site and its vicinity in GPR142, and implicit membrane was used in induced fit docking. All the ligands were prepared using LigPrep (Schrödinger Release, 2015) and were optimized with OPLS force field. The induced fit docking was carried out in different stages. During first stage, ligands were docked to rigid protein using initial softened-potential Glide docking with vdW van der Waals radii scaling of 0.7/0.5 for receptor/ligand, respectively. The top 20 poses for each test ligand were used to sample protein plasticity using Prime module of Schrodinger suite software (Jacobson et al., 2002, 2004). In the next step, receptor sampling and refinement was performed. Residues having at least one atom within 5 Å of any of the 20 ligand poses were subjected to a conformational search and minimization while residues outside this range were fixed. So, in this way the flexibility of proteins was taken into account. The backbone, side-chains and ligand were subjected to subsequent energy minimizations. Further, re-docking of the ligands was carried out into their respective 10 structures that were selected within 30.0 kcal/mol of their lowest energy structure. Glide XP (extra precision) was used for all the docking calculations. Finally, ligand poses were scored using a combination of Glide Score functions and Prime (Jacobson et al., 2002, 2004) where the top ranked poses for each ligand were chosen as their respective final results.

#### Pharmacophore development

The common pharmacophore hypothesis was performed using Phase module of Schrödinger software suite (Dixon et al., 2006a). Sixty compounds had been reported as potential GPR142 agonists (Du et al., 2012; Lizarzaburu et al., 2012) and compounds with EC_50_ value range between 0.036 and 33.00, were selected from literature. These compounds were prepared by generation of stereoisomers, neutralizing the charges on the structures and generating ionization states at pH 7.0 using OPLS2005 force fields. One thousand conformers were generated per compound by ConfGen module of Schrodinger suite software (Watts et al., 2010). All the conformations were pre-minimized and post-minimized. OPLS2005 force field was used with GB/SA water solvent treatment for calculation of minimization. Distance dependent dielectric and maximum relative energy difference were 10.0 Kcal/mol relative to the global energy minima and redundant conformers were eliminated.

The Pharmacophore features i.e., Acceptor (A), Donor (D), Hydrophobic (H), Negative (N), Positive (P), and Aromatic Rings (R) defined by three chemical structure patterns were point, vector and groups as SMARTS queries. These patterns were assigned as one of three possible geometries, which defined the physical characteristics of site. In case of aromatic rings, the site includes directionality, defined by a vector that is normal to plane of the ring.

A scoring function was used to examine the common pharmacophore features (CPHs) to yield the best alignment of active ligands and quality of alignment measured by a survival score defined as:

$$\begin{array}{l} S=W_{\text{site}}S_{\text{site}}+W_{\text{vec}}S_{\text{vec}}+W_{\text{vol}}S_{\text{vol}}+W_{\text{sel}}S_{\text{sel}}+W_{\text{rew}}^{\text{m}} \end{array}$$

where weights are represented by W_Ds_ and scores are represented by S_Ds_, and S_site_ represents an alignment score, S_vec_ represents the vector score and averages the cosine of the angles formed by corresponding pairs of vector features in aligned structures. S_vol_ represents the volume score based on overlap of the van der waals models of non-hydrogen atoms in each pair of structures. S_sel_ represents the selectivity score, and accounts for the fractions of molecules that are likely to match the hypothesis regardless of their activity toward a receptor. W_site_, W_vec_, W_vol_, and W_rew_ have a default value of 1.0 while W_sel_ has a default value of 0.0. ${\text{W}}_{\text{rew}}^{\text{m}}$ represents the reward weights, where m is the number of actives that match the hypothesis minus one.

Different data sets were used to build the pharmacophore features:

##### First data set (high affinity EC_50_ value)

All the 60 compounds were chosen. The activity threshold was set to 0.036. The compounds were considered active above 0.036 and inactive below 0.001. The maximum activity was at 0.930 and minimum activity was 0.036. The hypothesis was selected to match at least 35 compounds out of 38 actives.

##### Second data set (medium affinity EC_50_ value)

All the 60 compounds were in second Dataset and activity thresholds were set in such a way that, the compounds are active if activity was above 1.060 and inactive it was below 1.000. The maximum activity was set at 6.600 and minimum activity was fixed at 1.060. The hypothesis was set to match at least 30 compounds out of 51 actives or active group.

##### Third data set (low affinity EC_50_ value)

All the 60 compounds were chosen in the third Dataset and activity thresholds were set again in such a way that compounds are active if activity was above 0.036 and inactive if below 0.035. The maximum activity was at 33.000 and minimum activity at 0.036. The hypothesis was set to match at least 20 compounds out of 60 actives or active group.

The hypothesis generated was used for matching against screened ligands.

### Combining the ligand based virtual screening with structure based virtual screened compounds

A Phase database was created for the best compounds obtained from virtual screening from different compound libraries. A maximum of 100 conformers per structure of the phase database were generated and up to 10 conformations per rotable bonds were retained. This database was then searched to match the pharmacophore hypothesis ADPRR. The Phase database was searched for geometric arrangements of pharmacophore sites that match inter-site distances and the site types. The conformers that aligned to the hypothesis were rapidly retrieved from the database. Fitness score was used to filter the conformers or hits and then filtered by number. A comparative analysis was done for experimental compound (with known EC_50_ value 0.036) with compound21 obtained from matching with pharmacophoric hypothesis.

### Biochemical pathway construction of GPR142 complexes with the potential compound

In order to explore the signal transduction in cellular process of GPR142 membrane protein that terminates with the regulation of transcription or downstream cellular process and ultimately to understand their effect on insulin secretion, a biochemical pathway for the GPR142 was constructed in presence of potential compound. The interacting species (gene, protein, and other molecules) were prioritized, collected from different sources and literature survey, that included association studies of GPR142 with drug or drug like compounds, linkage studies of GPR142 and GPR41, gene expression studies related to insulin production, drug kinetics in diabetes, and biological regulatory pathways of type 2 diabetes. The concentrations were assigned for each gene, protein and other molecules in micro molar. A mathematical computational model of the signaling pathway of GPR142 was then developed and visualized in Cell Designer v4.4 (Funahashi et al., 2003). Systems biology approach was used to investigate interactions of ligands with known EC_50_ vlaues (0.054) and different concentrations of virtual screened ligands against the GPR142. GPR142, GPR41, Gαs, Gαi, Gαq/11, and Gα12/13 data were retrieved from different databases, servers, tools and literature (Kanehisa, 1996, 1997, 2002; Kanehisa and Goto, 2000; Kanehisa et al., 2002, 2004, 2006, 2008, 2010, 2011, 2014; Moriya et al., 2007; Harmar et al., 2009; Kotera et al., 2012; Muto et al., 2013; Nakaya et al., 2013). A complete GPR142 pathway beginning with potential compound binding to GPR142 for Type 2 diabetes was constructed within cell compartment. During simulation the input values were assigned using kinetic irreversible simple Michaelis Menten equatio$n\;V=\frac{VmS}{Km}+S$ and mass action kinetics equation *V* = *K Π*i S. The kinetic simulations were used to investigate which genes and proteins interact with each other and effect the insulin secretion.

### Molecular dynamics simulation

Molecular dynamics (MD) simulation was performed using Desmond package v31023 (Bowers et al., 2006; Guo et al., 2010; Shivakumar et al., 2010). MD simulation of GPR142 complex structure with compound2 and compound21 was performed by Desmond Schrödinger package for 50 ns (nanoseconds) each. The system was build using simple point charge water (SPC) model with membrane model 1-hexadecanoyl-2–(9Z-octadecenoyl)-*sn*-glycero-3-phosphocholine (POPC) by applying periodic boundary conditions (PBC) in simulation box (orthorhombic). An embedded system neutralized with counter ions and geometry of SPC molecules, SHAKE algorithm neutralizing heavy atom bond lengths with hydrogen's and particle mesh ewald (PME) were applied for electrostatic interactions. The full system composed of GPR142 structure with compound2 and compound21 was simulated through multistep MD simulation protocols, where initially system was minimized with restraints on solute for maximum 2,000 iterations using steepest descent and followed by conjugate gradient algorithm with 50.0 kcal/mol/Å threshold energy. The system equilibrations were performed by applying 10 ps (picoseconds) simulation time for non-hydrogen solute atoms in NVT ensemble at 10 K temperature. Then 12 ps MD simulations were performed for restraining non-hydrogen's solute atoms in the NPT ensemble at 10 K temperature. Further, 24 ps MD simulation were performed for restraining all non-hydrogen solute atoms in the NPT ensemble at 300 K temperature and similarly 24 ps MD simulation were again performed to relax the system without restraints in the NPT ensemble at 300 K temperature. Complex structure of GPR142 with compound2 and compound21, 50 ns each MD simulations were performed. Trajectories were recorded after every 4.8 ps, where energy recording interval was 1.2 ps. RMSD and RMSF of the complex structure of GPR142 with compound2 and compound21 in each trajectory was analyzed with respect to 50 ns simulation using OPLS2005 (Optimized Kanhesia for Liquid Simulations) force fields.

## Results

### Structural modeling and validation of 3D-model of GPR142

The 3D-structure of GPR142 was modeled using threading and *ab initio* method. Ab initio approach is based on the “thermodynamic hypothesis,” which states that the native structure of a protein is the one for which the free energy achieves the global minimum; ab initio is most difficult approach, but a very useful approach. Threading and ab initio/*de-novo* approach were applied to predict the 3D structure of GPR142 using available structural information from the resolved X-Ray structures in PDB databank. Out of 462 amino acids, 283 were modeled from residue 151-433. Residues 1-150 from the N-terminus and 434-462 from the C-terminus were trimmed (Kaushik and Sahi, 2017). A total of twenty models were generated and validated by SAVE server (Kaushik and Sahi, 2017). Best predicted model structures were further refined by using Modeler v9.8, calculation of probability density function (pdfs) and Discrete optimized potential energy (DOPE). The 3D-model had DOPE score of −34453.63 which was lowest against the predicted other models. Also, Ramachandran plot showed 94.9% residues in the allowed region that depicted the stability of predicted model.

### Active site prediction

The top ranked potential receptor binding sites were identified using SiteMap. The best site had a score of 1.12 Å, 521 Å^3^ volume, 0.72 hydrogen bond acceptor score, 0.68 hydrophilic score, and 1.00 hydrophobic score. The active site residues were identified as Phe212, Arg224, Asn235, Glu238, Trp300, Arg301, Lys314, and Asp397. Active site regions were largely located in extracellular regions of seven transmembrane domains where the potential leads can bind and play crucial role in signal transduction.

### 3D tunnel representation and domain prediction

**Figure 2:**
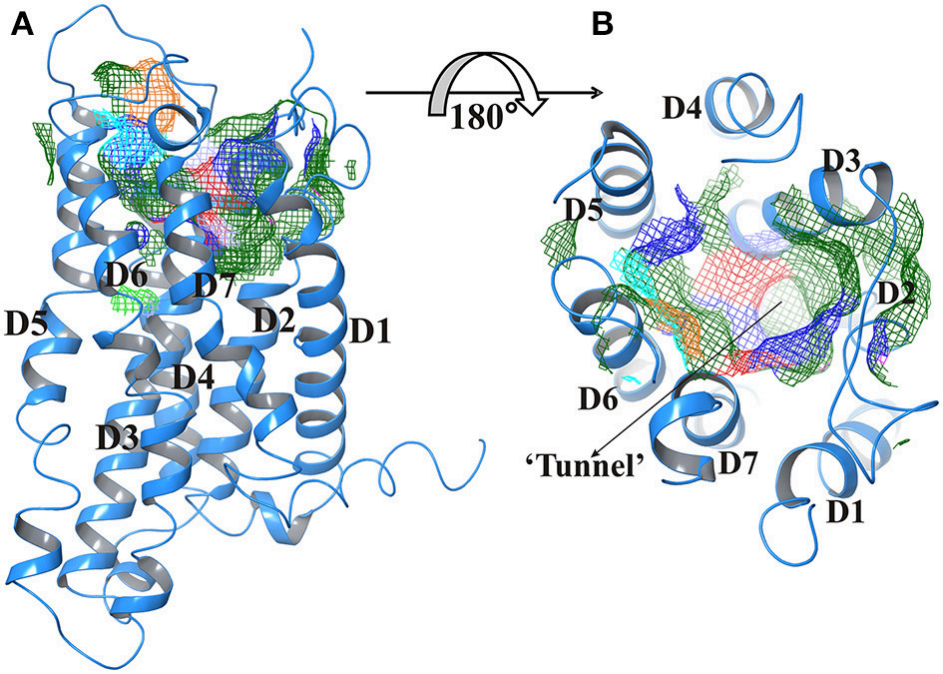
**(A)** Predicted active site of GPR142 using Sitemap showing different domains. (**B)** Tunnel region of GPR142; where active site resides (Surface in mesh form) are color coded according to their property like hydrophobicity, charge, and polarity. The Orange color depicts GLY and atoms without PDB residues name, Green, Hydrophobic residues; Cyan, Polar uncharged residues; Blue, Positively charged residues, and Red, Negatively charged residues.

Figure 2 represents the Trans membrane domains: 160 to 182, TM2 194 to 216, TM3 236 to 258, TM4 278 to 300, TM5 315 to 337, TM6 357 to 379, and TM7 394 to 416. The tunnels were generated using CAVER2.1 program. The tunnel leading to active site had following coordinates; X coordinate at −4.95, Y coordinate at −70.93, and Z coordinate at 66.58. Whereas, bottleneck radius, length, curvature average gate radius was 3.60, 12.01, 1.03, and 4.67, respectively.

### Receptor based virtual screening

A total of 1171519 compounds obtained from different libraries were docked into the predicted active site of GPR142. A step wise filtering protocol was used, in the first stage compounds were docked using HTVS where a total of 112,927 hits were obtained. These 112,927 compounds were further docked with Glide SP where a total of 11,281 hits were obtained. Finally, the hits from previous stage were subjected to Glide XP docking and only one pose per ligand was retained. Finally, a total of 1,120 hits were obtained as shown in Table 1.

**Table 1:** Combinatorial library of chemical compounds which represents the number of input compounds in different parameters of Glide Schrodinger suite software and number of outputs compounds.

**Zinc**	**358399**	**35831**	**3576**	**349**	**349**
SchrodingerDB	416151	41615	4161	416	415
TimTec	9211	921	92	09	09
PUBCHEM	21592	2159	215	21	20
Not annotated NCI	17487	1748	174	17	01
Marine	419	41	04	01	01
DrugBank	5352	532	52	08	08
ChemBank	3919	391	39	3	03
Anti HIV NCI	10100	1010	101	10	10
Drug Likness NCI	200052	20005	2000	200	200
Asinex Ltd.	86748	8674	867	86	86
ChEBI	42089	0	0	0	0
Total	1171519	112927	11281	1120	1120

*Top 20 screened compounds were shortlisted on the basis of docking scores (range −13.041 to −8.0)*.

**Figure 3:**
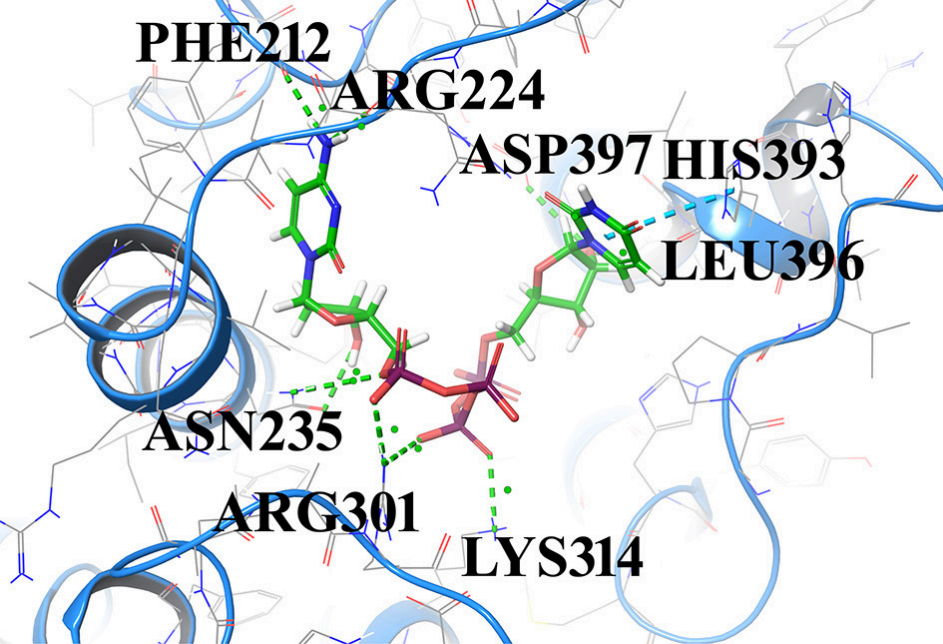
Compounds1 docked in the active of GPR142. The compound is shown in sticks and the protein is depicted as ribbons and H-bonding is shown as dotted lines.

Compound1 with a docking score of −13.041 is a comfortable legend in the active site of GPR142. The 2,4-dioxo-(1,2,3,4-tetrahydropyrimidin-1-yl)-3,4-dihydroxyoxolan-2-yl moiety forms hydrogen bond with side chain residues Arg224 and Asp397. The oxy phosphinato moieties form strong H-bond interactions with side chains residues Asn235, Arg301, and Lys314. The amino group of the dihydropyrimidine moiety forms H-bond with the backbone oxygen of residues Phe212 and Arg224 (Figure 3). The interactions analysis for the 20 screened compounds is given in the Table 2 and their 2D structures are given in Supplementary Data (Table S3).

**Table 2:** Top 20 screened chemical compounds screened based on using virtual screening along with respective Glide emodel score, number of Glide poses, their hydrogen bond interactions, and ADMET properties.

**Compound's**	**Molecular formula**	**Database's**	**Docking score**	**Glide emodel**	**Glide poses**	**Interactions (Hydrogen bond interactions)**	**logP**	**logS**
Compound 1	C18H23N5O21P4	DrugBank	−13.041	−93.629	40	Arg301, Asn235, Phe212, Arg224, Asp397, Lys314	−0.53	−1.34
Compound 2	C27H30O16	ChemBank	−11.331	−88.517	1	Arg224, Leu396, Asp397, His393, Asp389	0.15	−2.24
Compound 3	C28H32O15	ChemBank	−11.265	−90.187	5	Arg301, Asn235, Lys314, Gln225	0.08	−2.60
Compound 4	C19H26I3N3O9	DrugBank	−10.694	−78.884	32	Asp389, His393, Asn400, Arg224, Phe212	−2.78	−2.66
Compound 5	C21H26N7O14P2	ChemBank	−10.433	−93.381	1	Asp389, His393, Leu222, Arg224, Lys314, Arg301, Ala213, Ala234, Asn235	−1.38	−2.22
Compound 6	C41H42O6	DrugBank	−8.971	−107.43	2	Arg224	6.11	−6.24
Compound 7	C29H30N6O4S1	Zinc	−8.743	−74.994	11	Leu396, Arg301	4.57	−5.07
Compound 8	C30H35N3O2	Zinc	−8.620	−74.959	46	Asp397	−0.05	−8.53
Compound 9	C20H21N1O8P1	Druglikness	−8.609	−74.756	1	Arg301, Asn235, Lys314	1.16	−4.10
Compound 10	C22H30N4O6	AntiHIV NCI	−8.530	−83.930	1	Arg301, Asn235, Arg224, Glu238, Asn242	0.23	−4.76
Compound 11	C21H24N2O5P1	Druglikness	−8.492	−63.345	2	Lys314, Arg301, Arg373, Asn400	2.60	−4.06
Compound 12	C22H19Cl3O5	Druglikness	−8.464	−67.858	5	His380, Lys314	4.31	−5.33
Compound 13	C12H20N3O7	PUBCHEM	−8.386	−43.623	7	Asn400, Asn242	−2.00	−0.95
Compound 14	C29H28F1N5O4S1	Druglikness	−8.301	−81.932	3	Asp389, Leu396, His393, Asp397, Arg224	3.47	−5.73
Compound 15	C41H32O11	Zinc	−8.281	−82.301	2	Asn400, Val231	4.80	−5.57
Compound 16	C20H21N1O8P1	Schrodinger	−8.194	−68.208	9	Arg301, Lys314, Asn235	1.16	−4.10
Compound 17	C33H32N4O8	Zinc	−8.098	−93.738	1	Arg224, Arg373, Asn400	3.61	−5.52
Compound 18	C24H34N4O6	Druglikness	−8.089	−80.004	18	Asn235, Glu238, Asn242, Asp389, His393	0.53	−5.15
Compound 19	C26H38N2O4	Druglikness	−8.050	−52.984	1	Arg301	3.69	−5.55
Compound 20	C23H19NO10	Druglikness	−8.036	−58.247	1	Asn235, Arg301, Arg373, Asn400	3.27	−4.73

Compound2 (docking score −11.331) and compound3 (docking score −11.265) both have chrome-4-one group and trihydroxy methyl oxanyl moieties. In case of Compound2 the hydroxyl groups form strong H-bond interactions with Arg224 and Asp397 residues. The hydroxy groups of dihydroxyl phenyl moiety form strong interactions with Leu396 and His393. The Chrome-4-one moiety is oriented in such a way that it has hydrophobic interactions with Ala213, Leu394, and Met377.

In compound3 although it fitted well in the active site, however, the orientation was such that the hydroxy methoxy phenyl moiety did not have any H-bond interactions. Compound4 and compound14 are dicarboxamide derivatives. In Compound4, the dihydroxy propyl amino group had H-bonding interaction with Asn400 and Arg224 residues. Hydrophobic interactions were observed with Ala213, Val226, Ala234, Asn235, Leu394, Leu396, and Asp397 residues. In compound14, the carboxamide moiety interacted with Leu396 and His393 residues. The amino group attached to the thiazole ring had H-bond interaction with Arg224 and Asp397 residues. Compound5 (docking score −10.076) was structurally similar to Compound1. The phosphonate oxy groups formed strong H-bond interactions with side chain of Lys314 and Arg301 residues. The purine moiety formed H-bond with Ala213 residue and the carbamoyl moiety of pyridine had H-bonded interaction with Leu222 and Arg224 residues. Compound6 with docking score of −8.97 formed H-bond with Arg224 residue. It had strong hydrophobic interactions with Asp397, Leu396, Asn235, Ala234, Glu238, and Lys314 residues. Compound7 (docking score of −8.743) sits well in the cavity of GPR142. The indole triazine moiety has hydrophobic interactions with Phe212, Ala213, Ala234, Val226, Asp397, and Asn400 residues. The oxygen of imidazole ring forms H-bond with Leu396 residue. The oxygen of hexonoate group has strong H-bond with Arg301 residue. Compound8 well occupied the binding site of GPR142 through hydrophobic interactions. Only one H-bond was observed between aminogroup of benzylamino moiety and side chain of Asp397 residue. Compound9, compound11, and compound16 are derivatives of phosphonic acid. In all the three compounds, the phoshonic acid moiety formed H-bond with side chain of Arg301 and Lys314 residues. The oxygen of carbamoyl moiety formed H-bond with side chain of Asn235 in compound9, compound16 and Asn400 with compound11. Hydrophobic interactions were also observed with Val209, Val226, Ala234, Asn235, and Leu396. Compound10 and compound18 both have anthracene group. In compound10, hydroxyl ethyl amino group formed H-bond with Arg301, Asn235, Glu238, and Asn242 residues. Hydrophobic interactions were observed with Ala234 and Val226 residues; whereas, in compound18 the hydroxyl propyl amino group formed H-bond with His393 and Glu238 residues. The hydroxyl group attached to anthracene moiety showed H-bonding with Asn235 residue. Compound12 had mainly hydrophobic interactions and one hydrogen bond. The hydroxyl methyl group formed H-bond with Lys314 residue. Hydrophobic interactions were observed with Ala234, Asn235, and Glu238 residues. In compound13, hydroxyl group of pentahydroxy hexyl imino group formed H-bond with Asn242 and Asn400 residues. Hydrophobic interactions were observed with Ile206, Val209, Ala234, Arg224, and Ala213 residues. Compound15 had hydrophobic interactions with Phe212, Arg224, Ala234, Val231, Asn235, Leu396, His380, Asn400, and Asp397 residues. It did not show any strong H-bonds. Compound17 had strong H-bond interaction between with side chain of Arg224 and Arg373 residue. Hydrophobic interactions were observed with Asp397, Leu396, and His317 residues. In compound19, hydroxyl group of the phenyl moiety formed H-bond with side chain of Arg301 residue. The major hydrophobic interactions were with residues Ala213, Ala234, Asn235, Phe239, Lys314, Glu238, and Leu396. In compound20, hydroxyl group formed H-bonds with Asn235, Arg301, and Arg373 residues. Hydrophobic interactions were observed with Ala234, Glu238, and Leu396 residues.

Strong hydrogen bond interactions with amino acid residues Arg224, Asn235, Arg301, Lys314, Asn85, and Asp397 played a key role in binding affinity of potential compounds with GPR142. Therefore, compounds with donor or acceptor groups that can form H-bonds with these residues are likely to have better affinity.

### Validation

#### Blind docking

**Figure 4:**
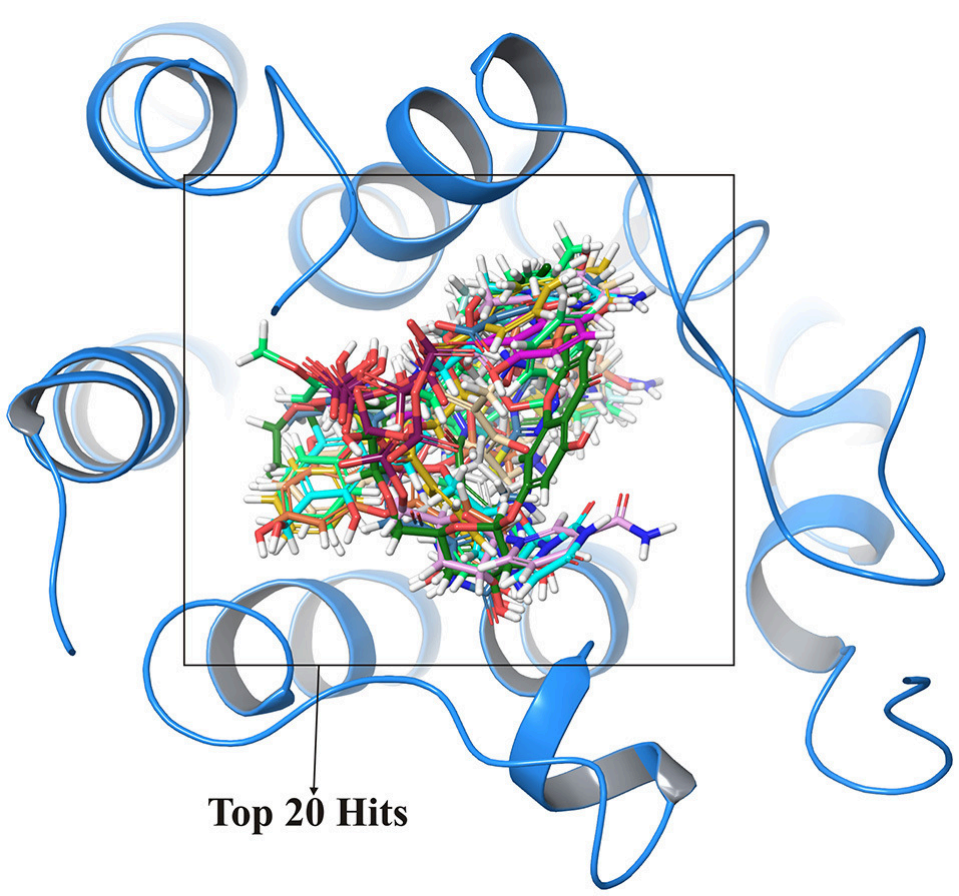
Cross validation of active site region where top 20 compounds shown in different colors were docked at same active site regions which validate the structural activity.

In order to cross validate the above results blind docking for top compounds was performed. All the compounds docked in the active site region are shown in Figure 4 and hence, eliminating the possibility of other binding sites for these screened compounds.

#### Induced fit docking

**Figure 5:**
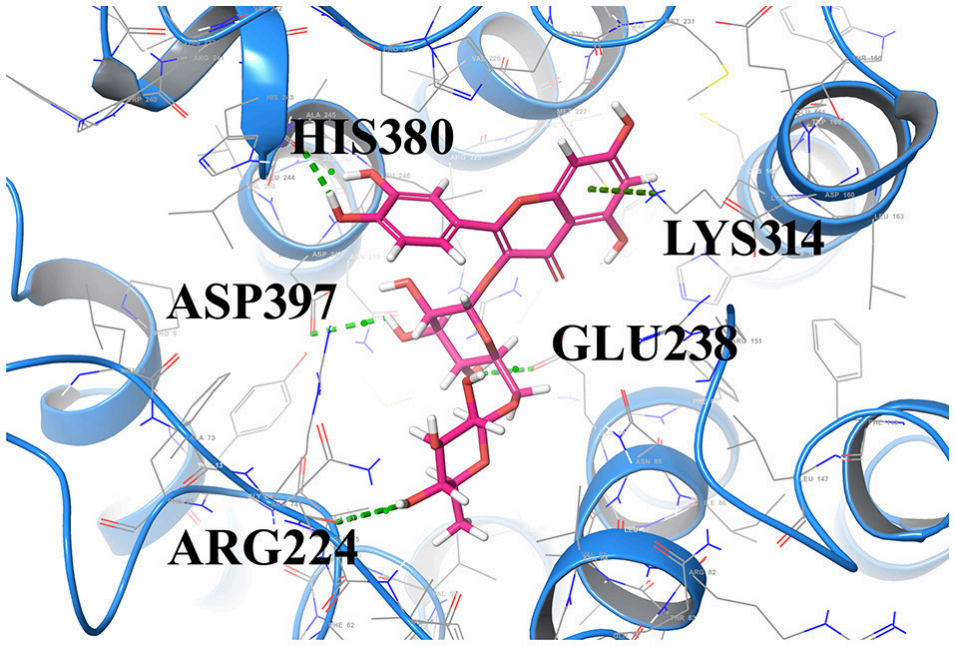
Compound2 with the highest Induced Fit docking score after docking at same active site, where receptor and ligand both were flexible. Interacting residues are shown as sticks. The dotted lines respresent the H-bonded interactions.

The most important feature of induced-fit docking (IFD) is that both ligand and the residues in receptor's active site and its vicinity are imparted flexibility. The results of IFD for the 10 screened compound was done to validate and refine the interactions (Table 3). The Induced fit docking of compound2 with GPR142 (Figure 5) showed with best docking score of −13.449 and strong H-bonds. Therefore, compound2 was selected for further studies.

**Table 3:** Top 10 potential screened chemical compounds after Induced Fit docking analysis showing the score and the non-bonded interactions.

**Compound's**	**Induced fit (XP) docking**	**IFD score**	**XP interactions**
Compound1	−11.173	−577.105	Arg301, Trp300, Asp397, Asn235, Glu238, Lys314
Compound2	−13.449	−572.7	Arg224, Lys314, Leu396, His380, Asp397, His393, Asp389
Compound3	−11.567	−569.821	Asn235, Leu396, Asp397, Arg224, His380, Leu222, Asp389, His393
Compound4	−9.6643	−572.504	Ile206, Glu238, Arg373, Asn400, Arg224, Tyr322
Compound5	−12.3708	−574.37	Val383, Lys314, His380, Arg301, Asn235, Gln225, Leu222, Leu396, His393, Asp389
Compound6	−10.468	2828552	–
Compound7	−9.1587	969.8008	His380, Ala395, Leu396, Asp397
Compound8	−10.600	−575.545	Glu238
Compound9	−9.7384	−572.791	Lys314, Arg301, Asn235
Compound10	−10.170	−565.303	Trp370, Arg373, Asn400, Asp397, His393, Asp389

#### Common pharmacophore hypotheses generation

**Figure 6:**
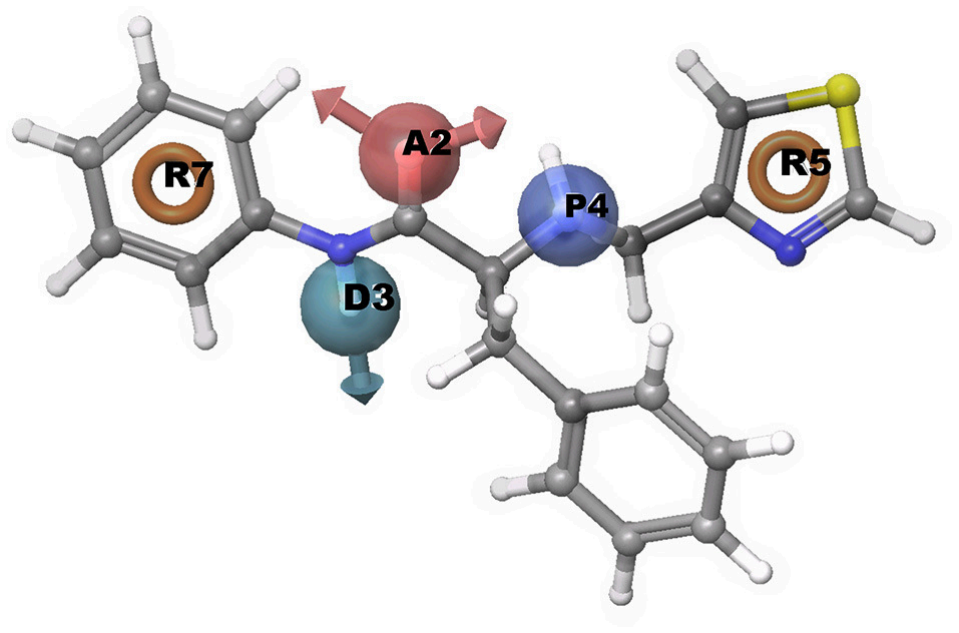
Representation of common pharmacophore hypotheses, where R5 has most important common pharmacophoric feature required to inhibit Type 2 diabetes.

A common pharmacophore hypothesis was generated using Phase module of Schrodinger suite software. The known experimental EC_50_ values for chemical compounds was retrieved from the literature. For information of experimental compounds used in study see Supplementary Data (Table S1). Using selected variants, the common pharmacophore hypothesis was generated amongst the given active ligands (Table 4). For scoring, the maximum and minimum number of sites were set at 7 and 4, respectively with a threshold such that at least 30 compounds should match out of 51 actives. Clustering was done to score hypotheses, vector and site filtering to retain those with RMSD below 1.20 Å and vector score above 0.50 Å. The best score hypotheses was ADPRR (Figure 6) with 3.224 survival score, 0.71 site score, 0.912 vector score, 0.604 volume of pharmacophoric feature. Survival score was calculated using survival score formula (1.000 vector score, plus 1.000 site score, plus 1.000 volume score, minus 0.000 reference ligand relative conformational energy, plus 0.000 selective score, plus 1.000 number of matches, plus 0.000 reference ligand activity).

**Table 4:** Selected variants for common pharmacophore hypothesis.

**Variant**	**Maximum number of hypotheses**
ADRRR	1
AADPR	5
AAPRR	8
APRRR	7
DPRRR	6
ADPRR	22
AADRR	3

#### Pharmacophore matching in screened compounds

**Figure 7:**
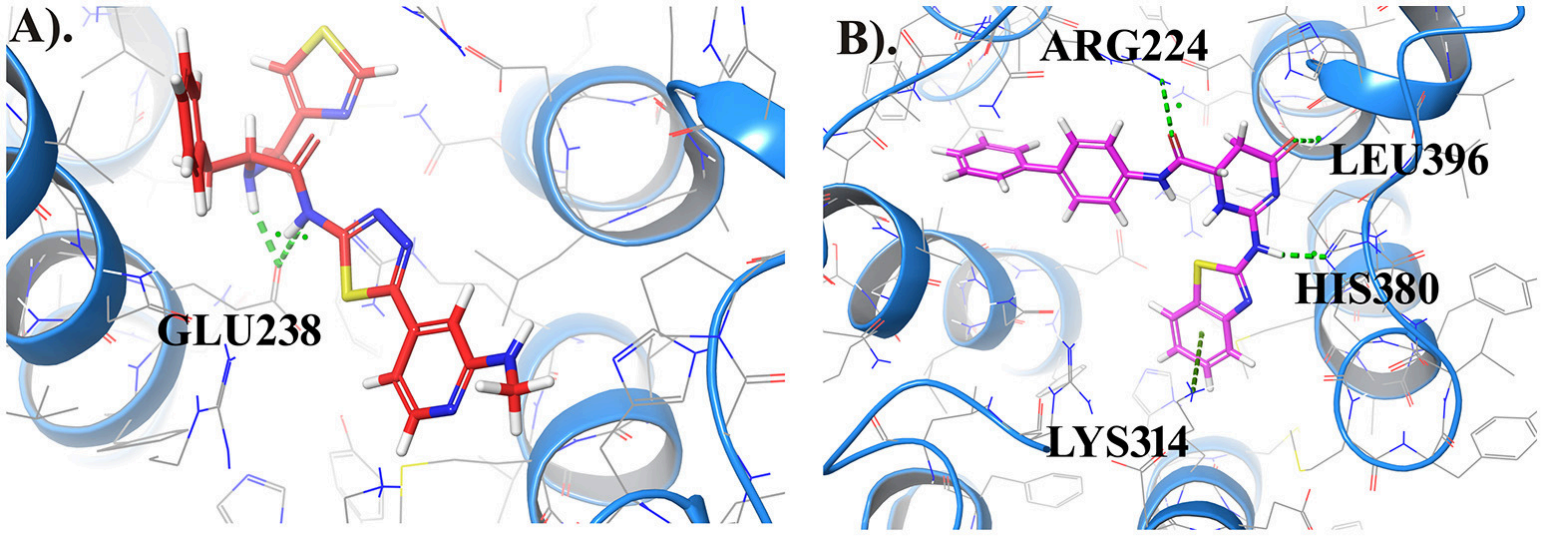
**(A)** Represents the docked complex of GPR142 with CompoundE1 (Experimental compound) and **(B)** Represents the docking complex of GPR142 with compound21. Hydrogen bonded interactions are shown as dotted lines. For clarity, only few transmembrane domains of the protein are shown.

Further, to investigate whether some of the screened compounds shared the pharmacophoric features derived from known potential GPR142 agonists, 1038 compounds obtained through receptor based virtual screening were searched for matches with pharmacophore hypothesis. Some of the screened compounds shared the same pharmacophoric features. Top 10 compounds showed ADPRR pharmacophoric features and their 2D structure are given in Supplementary Data (Table S2); where compound21 showed good binding affinity with the docking score of −6.470. Interestingly, Compound21-Compound 30 had good docking scores; however, comparatively the experimental compound (compoundE1) had better docking scores and stronger interactions. Comparative analysis between one of the compounds (CompoundE1) with EC_50_ 0.036 and compound21 revealed that both the compounds bound to the active site of GPR142. CompoundE1 had a docking score of −4.860 against that of −6.470 for compound21. Also, in CompoundE1 (mol wt: 450.56 g/mol) the amino group attached to the thiazole moiety formed H-bond with Glu238. It had hydrophobic interactions with Val204, Asn235, Ala234, Leu396, Asp397, where molecular weight of experimental compound was 450.568 g/mol shown in (Figure 7A). Compound21 (mol. Wt: 442.51 g/mol) had two strong H-bonded interactions with Arg224 and Leu396 residues. Hydrophobic interactions formed with Ala213, Val231, Ala234, Lys314, Met377, Leu394, Asp397, and His393 residues (Figure 7B). Using Pharmacophoric hypotheses approach new potential lead compounds were identified. ADMET analysis of compoundE1 showed logP value of 2.54 and logs value of −4.67 while compound21 showed good bioavailability of the compounds with oral absorption value of 93.391%, logP value 3.00 and logS value −5.88.

#### Biochemical pathway of GPR142 complexes with screened compound

**Figure 8:**
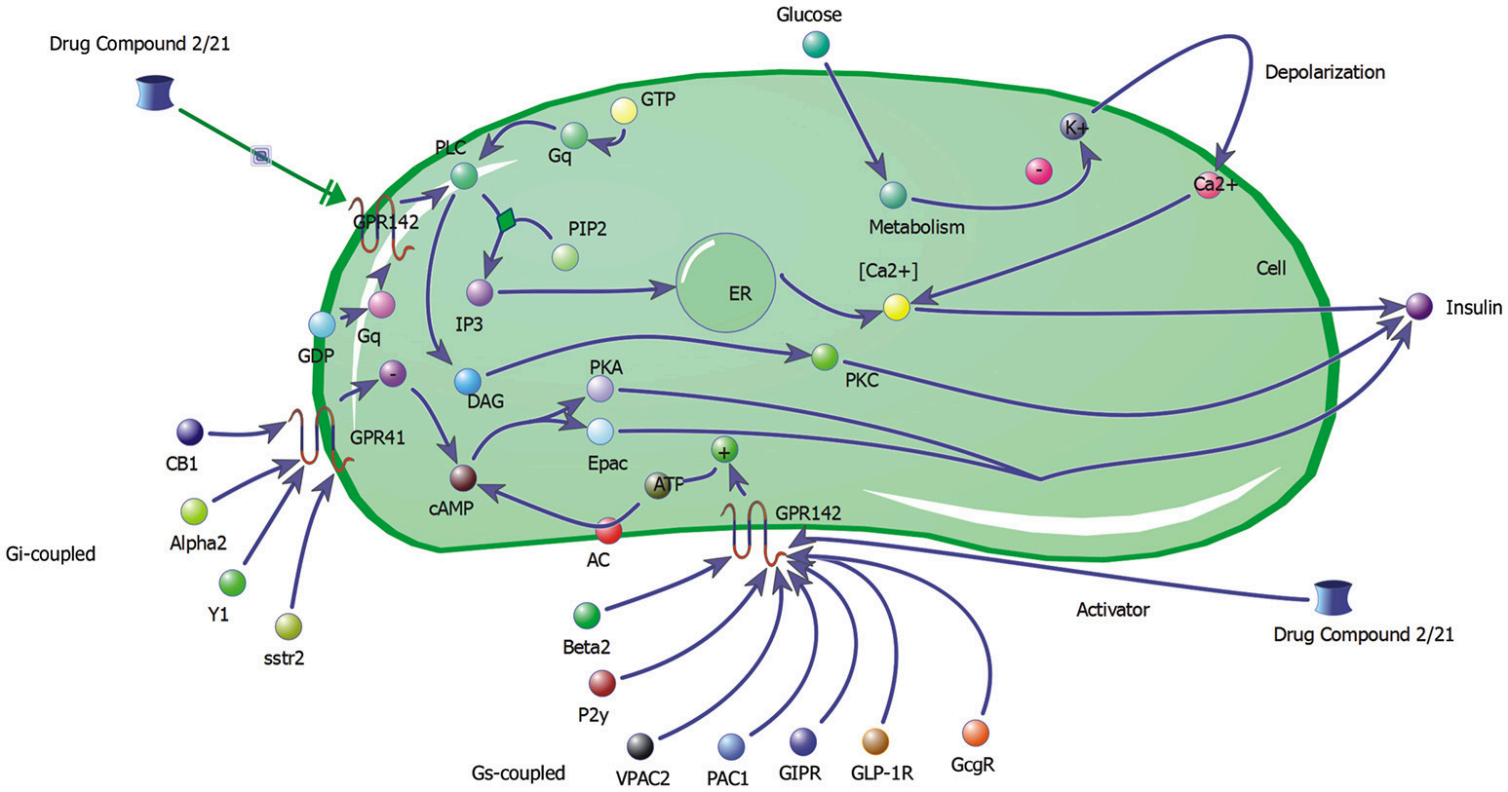
Network depicting three different signaling pathways through which insulin secretion may be enhanced on binding of compound2 and compound21 with GPR142.

**Figure 9:**
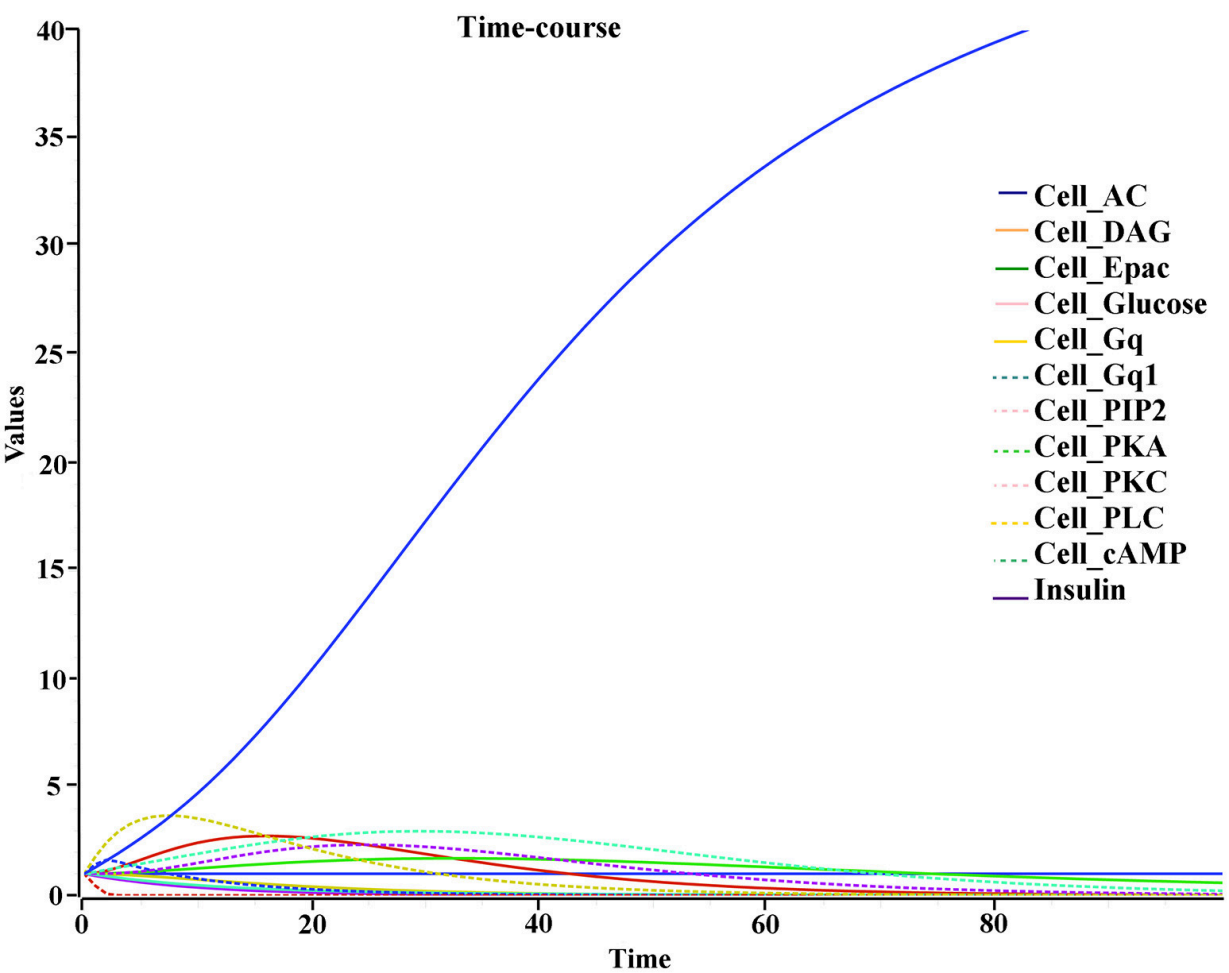
Kinetic studies of compound2 and its effect on insulin secretion X-axis represent the concentration of species and Y-axis represents the time of interaction.

The biochemical pathway of GPR142 complexes with compound2 and compound21 was constructed to study the effects of these compounds under the assumption that these compounds bind to GPR142, as shown by virtual screening, on the biochemical pathway in type 2 diabetes. In the network three different signaling pathways were identified through which insulin secretion enhanced on binding of compound2 and compound21 with GPR142 (Figure 8). Stimulation of GPR142 by diverse hormones, growth factors and compounds stimulate the hydrolysis of Phosphatidylinositol 4,5-bisphosphate (PIP2) by phospholipase C (PLC) and produces two second messenger as diacylglycerol (DAG) and inositol 1,4,5-trisphosphate (IP3) through activation of Gq signaling pathway. (1) DAG in turn stimulates protein kinase C (PKC) which triggers insulin secretion. These results agree with the experimental results that activation of Gq and Gi signaling by GPR142 agonists can stimulate glucose-dependent insulin secretion. (2) IP3 stimulates downstream signaling pathways and activates Ca^2+^ mobilization which may enhance insulin secretion. (3) GPR142 through Gi signaling pathway binds to adenyl cyclase (AC) and activates cAMP pathway which may regulate insulin secretion through protein kinase A (PKA) and exchange protein directly activated by cAMP (Epac). The kinetic simulations of the test compounds were done at different concentrations to see the effect on insulin production. The kinetic studies were carried out using different concentration of compounds. The optimum concentration which enhanced the insulin production was taken as 0.036 μM for compound2 (Figure 9). We previously published complete biochemical pathway of GPR142 network involved in type 2 diabetes (Kaushik and Sahi, 2015). The results showed significant increase in insulin production. However, an inhibitory effect was observed on cAMP production. This could be due to activation of GPR142 through Gq signaling pathway via DAG and IP3. The effect of different compounds/substrates/messengers on insulin and glucagon secretion is given in (Table 5) (Winzell and Ahrén, 2007).

**Table 5:** Hormones, ATP/ADP, ions and nutrients that affect insulin secretion via interaction through GPC.

**Ligand**	**Receptor**	**Effect on insulin secretion**	**Effect on glucagon secretion**	**G protein**	**Concentrations (mumol)**		
					**Value**	**Min**	**Max**
ATP/ADP	P_2*Y*_	Stimulatory	Not known	G_s_	1	0.1	1.9
CCK	CCK_*A*_	Stimulatory	Stimulatory	G_q_	1	0.1	1.9
Compound2	GPR142	Stimulatory	Not known	G_s_	0.036	0.036	0.068
Compound21	GPR142	Stimulatory	Not known	G_s_	0.036	0.036	0.068
Glucagon	Gcgr	Stimulatory	Stimulatory	G_s_, G_q_	1	0.1	1.9
GLP-1	GLP-1R	Stimulatory	Inhibitory	G_s_	1	0.1	1.9
GIP	GIPR	Stimulatory	Stimulatory	G_s_	1	0.1	1.9
NPY	Y_1_	Inhibitory	Stimulatory	G_i_	1	0.1	1.9
PACAP	PAC_1_	Stimulatory	Stimulatory	G_s_	1	0.1	1.9
Ach	Ca^2+^/K^+^	Stimulatory	Stimulatory	G_q_	1	0.1	1.9
AC	cAMP	Stimulatory	No Effect/ Modulating	G_i/o_	1	0.1	1.9
PLC	IP3/PIP2/DAG	Stimulatory	Inhibitory	G_i_	1	0.1	1.9
PKA	GPCR	Modulating Secretion	Inhibitory	G_q_	1	0.1	1.9
cAMP	Epac	Inhibitory	Stimulatory	G_i_	1	0.1	1.9
DAG analog	PKC	Stimulatory	Inhibitory	G_i_	1	0.1	1.9

#### Molecular dynamics simulation

Molecular dynamics (MD) simulations provided an insight into dynamic perturbations within the complex and interactions of ligand, lipid and water molecules.

**Figure 10:**
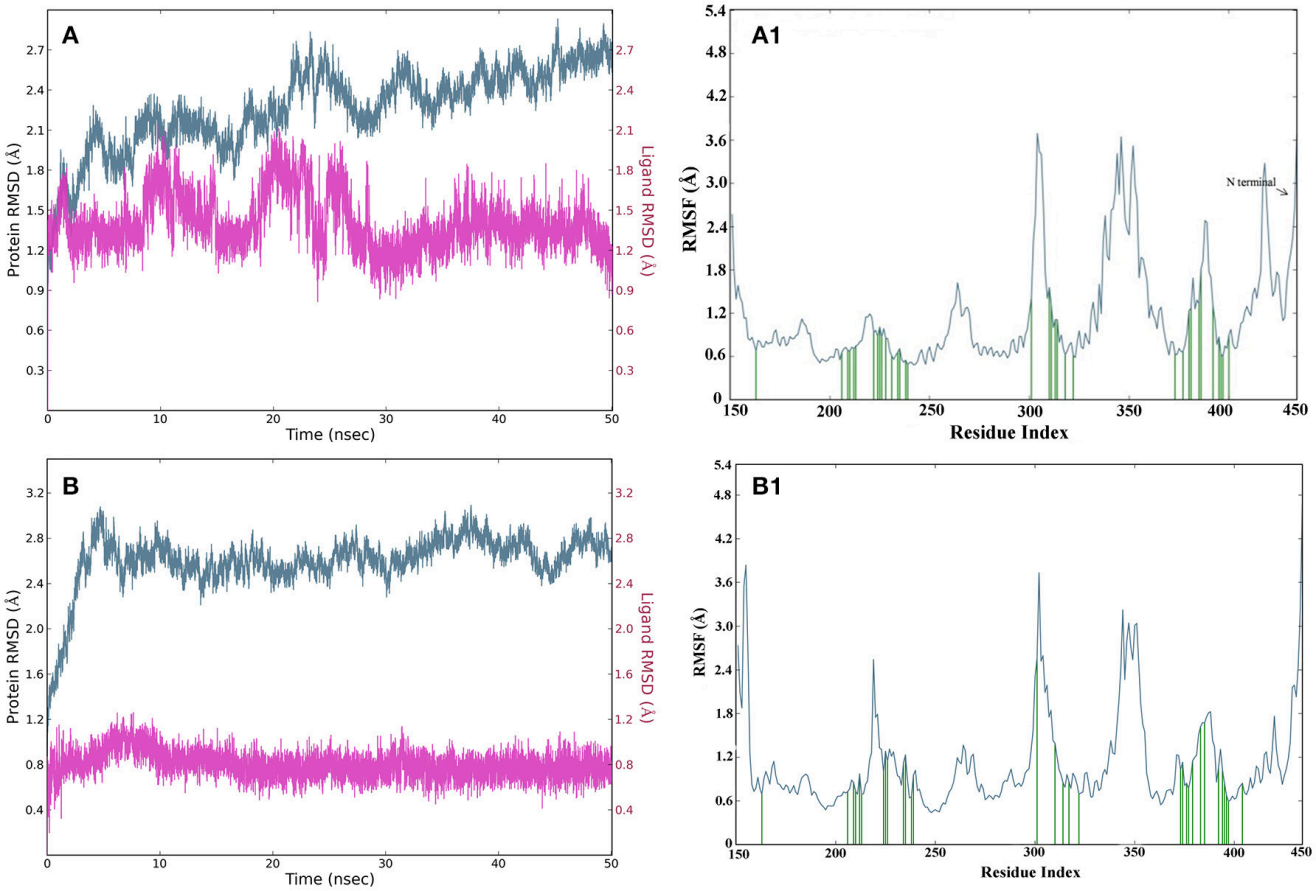
**(A)** RMSD of carbon alpha atoms of complex structure of compound2 for 50 ns simulation, where Y axis represents the RMSD value in Å and X axis represents the Time (ns) for GPR142 structure and compound21. **(B)** RMSD of carbon alpha atoms of complex structure of compound21 for 50 ns simulation, where Y axis represents the RMSD value in Å and X axis represents the Time (ns) of GPR142 structure and compound21. **(A1)** RMSF of carbon alpha of complex structure of compound2 for 50 ns simulation, where Y axis represents the RMSF value and X axis represents residues, where blue peaks represents the backbone, green peaks represents the ligand contacts. **(B1)** RMSF of carbon alpha of complex structure of compound21 for 50 ns simulation, where Y axis represents the RMSF value and X axis represents residues, where blue peaks represents the backbone, green peaks represents the ligand contacts.

The root mean square deviations (RMSD) of complex2 and complex21 protein were analyzed using carbon alpha (Cα) atoms and stability of compounds by using heavy atoms RMSD over 50 ns on generated 10430 trajectory frames. The complex2 protein Cα atoms RMSD showed between 1.8 and 2.7 Å while in complex21 showed higher RMSD but in constant range between 2.4 and 2.8 Å which is also comes in stable and acceptable range (Figures 10A,B). Compund2 heavy atoms showed stable and constant RMSD between 1.2 and 1.8 Å. It also showed some fluctuations in RMSD between 20 and 25 ns, after 25 ns it remain constant till end of the simulation. Compound21 also showed constant RMSD at 0.8 Å that means it was stable throughout simulation time.

The fluctuations in local domain of protein Cα atoms and effect of compounds binding in protein analyzed by root mean square fluctuations plot. Complex2 and 21 showed two higher fluctuations in loop regions, first fluctuation in loop which connects domain 3 and 4 between 300 and 312 residues and second connects domain 5 and 6 between 345 and 356 residues. N-terminal has large loop region between 421 and 450 residues with fluctuations in the acceptable range between 1.8 and 2.8 Å for the both complexes. Domain 3 and 4 loops did not show binding with compound2 while binding was recorded in compound21 (Figures 10A1,B1).

The interaction fraction analysis of ligand binding mode in protein based on occupancy of hydrogen and hydrophobic bonding throughout simulation periods. Compound2 showed more than 90% hydrogen boding with Asn235 and Glu238 while 20% with Arg224, His393, Asp397, and Asn400 residues. Hydrophobic occupancy showed between 30 and 60% during simulation with Val209, Phe212, Ala213, Val226, Met377, His380, Met381, Pro385, and Leu396 residues. In compound21, showed less than 20% hydrogen bonding with Arg224 and Leu396 while showed hydrophobic occupancy between 20 and 50% with the Val209, Ile210, Phe239, Lys314, Tyr322, Arg373, Met377, His380, Pro235 residues. These interaction fractions suggested that compound2 had strong binding affinity in comparison to compare compound21 as given in Supplementary Data (Figure S1).

## Conclusions

GPR142 is a potential drug target for diabetes. Using structure based virtual screening at the active site of GPR142, 1038 compounds were screened as potential inhibitors from the set of 1171519 compounds at different libraries. Further, top twenty screened compounds were selected and validated by blind docking and induced fit docking studies. The compounds that showed strong hydrogen bond interactions with amino acid residues Arg224, Asn235, Arg301, Lys314, and Asp397 were concluded as potential agonists of GPR142. Also, a pharmacophore hypothesis was generated using compounds with known EC50 values that searched against the screened compounds. A few compounds amongst the screened compounds shared the same pharmacophoric features as observed in the compounds reported in literature. The system biology approach was used to study the effect of compound2 and compound21 on insulin secretion. Interestingly both the compounds triggered insulin secretion on binding to GPR142 via Gq signaling pathway. Thus, we were able to identify structurally diverse compounds particularly compound1, compound2 and compound21 which can be used as scaffold to design and develop lead GPR142 agonists.

## Author contributions

AK and SS designed computational analyses. AK performed the analysis and network studies. AK, and SS analyzed the data and wrote the paper. All authors reviewed the manuscript.

### Conflict of interest statement

The authors declare that the research was conducted in the absence of any commercial or financial relationships that could be construed as a potential conflict of interest.
