# Supplementary material for: Structure Based Virtual Screening Studies to Identify Novel Potential Compounds for GPR142 and Their Relative Dynamic Analysis for Study of Type 2 Diabetes
Source: Front Chem. 2018 Feb 14;6:23. doi: 10.3389/fchem.2018.00023 (PMC5817085; doi:10.3389/fchem.2018.00023)
Supplement: Supplementary file 1 [file Table1.docx]

**Supplementary data**

**Structure Based Virtual Screening Studies to Identify Novel Potential Compounds for GPR142 and Their Relative Dynamic Analysis for Study of Type 2 Diabetes**

Aman Chandra Kaushik^1, 2^, Sanjay Kumar^3^, *Dong Quing Wei^1^ and *Shakti Sahi^2^

^1^State Key Laboratory of Microbial Metabolism and School of life Sciences and Biotechnology, Shanghai Jiao Tong University, Shanghai 200240, China

^2^School of Biotechnology, Gautam Buddha University, Greater Noida, U.P, India, 201312

^3^Molecular Structural Biology Division, CSIR-Central Drug Research Institute Lucknow, India

*Corresponding authors

**Table S1-** Experimental compounds obtained from literature [1-2], where table indicate EC50 value of chemical structure and structure

| Compounds’ | Structure | Molecular Formula | IUPAC Name | Experimental Activity (EC50) |
| --- | --- | --- | --- | --- |
| 01 | 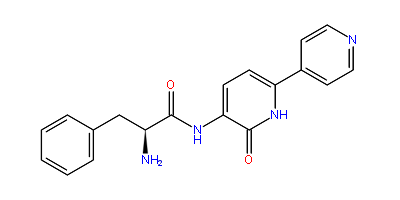 | C19H18N4O2 | (2S)-2-amino-N-[2-oxo-6-(pyridin-4-yl)-1,2-dihydropyridin-3-yl]-3-phenylpropanamide | 4.8 |
| 02 | 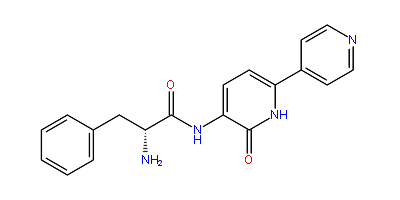 | C19H18N4O2 | (2R)-2-amino-N-[2-oxo-6-(pyridin-4-yl)-1,2-dihydropyridin-3-yl]-3-phenylpropanamide | 13 |
| 03 | 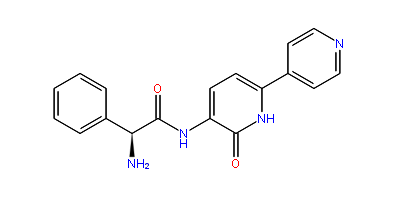 | C18H16N4O2 | (2S)-2-amino-N-[2-oxo-6-(pyridin-4-yl)-1,2-dihydropyridin-3-yl]-2-phenylacetamide | >33 |
| 04 | 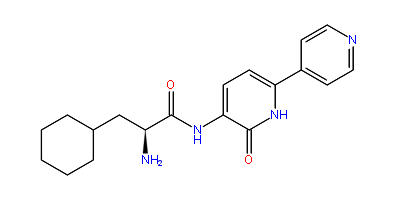 | C19H24N4O2 | (2S)-2-amino-3-cyclohexyl-N-[2-oxo-6-(pyridin-4-yl)-1,2-dihydropyridin-3-yl]propanamide | >33 |
| 05 | 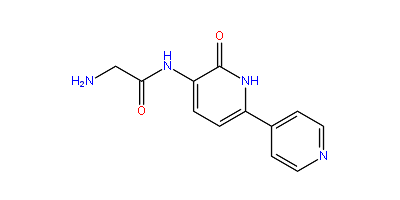 | C12H12N4O2 | 2-amino-N-[2-oxo-6-(pyridin-4-yl)-1,2-dihydropyridin-3-yl]acetamide | >33 |
| 06 | 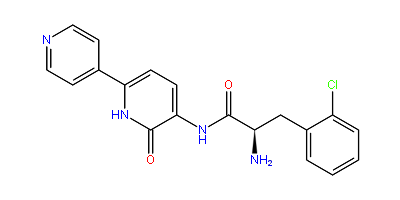 | C19H17ClN4O2 | (2R)-2-amino-3-(2-chlorophenyl)-N-[2-oxo-6-(pyridin-4-yl)-1,2-dihydropyridin-3-yl]propanamide | >33 |
| 07 | 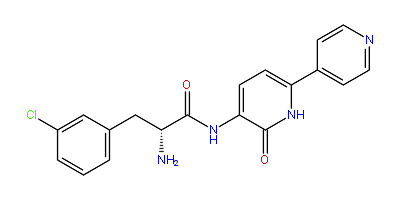 | C19H17ClN4O2 | (2R)-2-amino-3-(3-chlorophenyl)-N-[2-oxo-6-(pyridin-4-yl)-1,2-dihydropyridin-3-yl]propanamide | >33 |
| 08 | 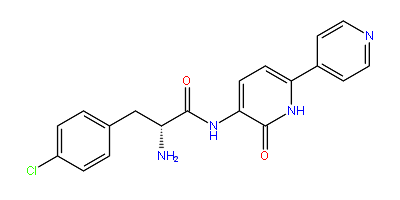 | C19H17ClN4O2 | (2R)-2-amino-3-(4-chlorophenyl)-N-[2-oxo-6-(pyridin-4-yl)-1,2-dihydropyridin-3-yl]propanamide | 3.8 |
| 09 | 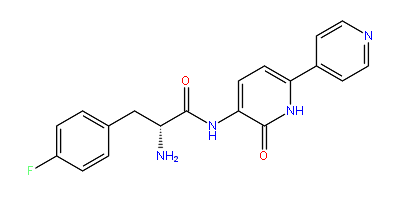 | C19H17FN4O2 | (2R)-2-amino-3-(4-fluorophenyl)-N-[2-oxo-6-(pyridin-4-yl)-1,2-dihydropyridin-3-yl]propanamide | 4.2 |
| 10 | 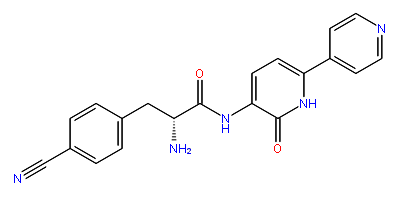 | C20H17N5O2 | (2R)-2-amino-3-(4-cyanophenyl)-N-[2-oxo-6-(pyridin-4-yl)-1,2-dihydropyridin-3-yl]propanamide | 4.6 |
| 11 | 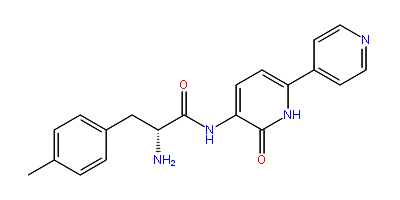 | C20H20N4O2 | (2R)-2-amino-3-(4-methylphenyl)-N-[2-oxo-6-(pyridin-4-yl)-1,2-dihydropyridin-3-yl]propanamide | 6.6 |
| 12 | 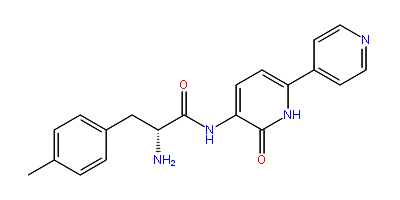 | C20H20N4O2 | (2R)-2-amino-3-(4-methylphenyl)-N-[2-oxo-6-(pyridin-4-yl)-1,2-dihydropyridin-3-yl]propanamide | 25 |
| 13 | 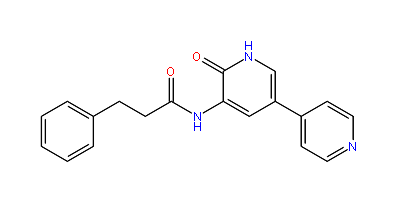 | C19H17N3O2 | N-[2-oxo-5-(pyridin-4-yl)-1,2-dihydropyridin-3-yl]-3-phenylpropanamide | >33 |
| 14 | 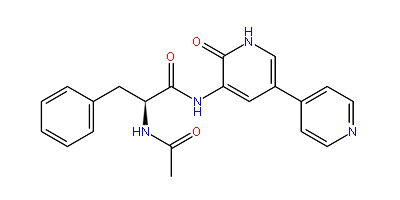 | C21H20N4O3 | (2S)-2-acetamido-N-[2-oxo-5-(pyridin-4-yl)-1,2-dihydropyridin-3-yl]-3-phenylpropanamide | >33 |
| 15 | 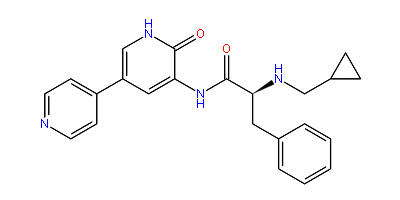 | C23H24N4O2 | (2S)-2-[(cyclopropylmethyl)amino]-N-[2-oxo-5-(pyridin-4-yl)-1,2-dihydropyridin-3-yl]-3-phenylpropanamide | 0.76 |
| 16 | 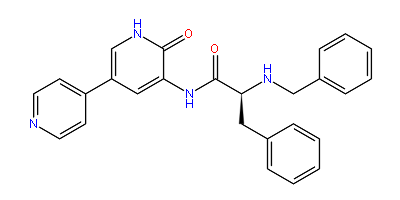 | C26H24N4O2 | (2S)-2-(benzylamino)-N-[2-oxo-5-(pyridin-4-yl)-1,2-dihydropyridin-3-yl]-3-phenylpropanamide | 0.83 |
| 17 | 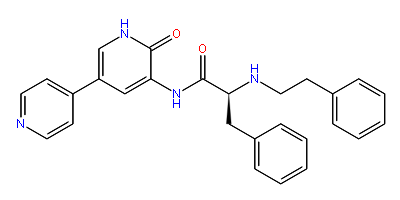 | C27H26N4O2 | (2S)-N-[2-oxo-5-(pyridin-4-yl)-1,2-dihydropyridin-3-yl]-3-phenyl-2-[(2-phenylethyl)amino]propanamide | 0.78 |
| 18 | 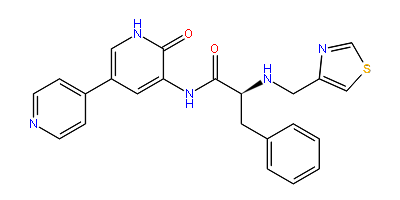 | C23H21N5O2S | (2S)-N-[2-oxo-5-(pyridin-4-yl)-1,2-dihydropyridin-3-yl]-3-phenyl-2-[(1,3-thiazol-4-ylmethyl)amino]propanamide | 0.093 |
| 19 | 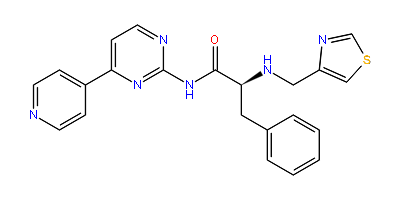 | C22H20N6OS | (2S)-3-phenyl-N-[4-(pyridin-4-yl)pyrimidin-2-yl]-2-[(1,3-thiazol-4-ylmethyl)amino]propanamide | 0.36 |
| 20 | 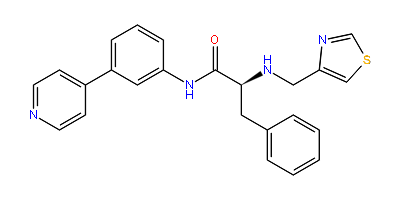 | C24H22N4OS | (2S)-3-phenyl-N-[3-(pyridin-4-yl)phenyl]-2-[(1,3-thiazol-4-ylmethyl)amino]propanamide | 0.088 |
| 21 | 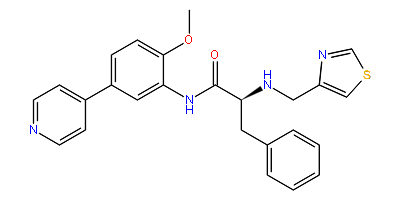 | C25H24N4O2S | (2S)-N-[2-methoxy-5-(pyridin-4-yl)phenyl]-3-phenyl-2-[(1,3-thiazol-4-ylmethyl)amino]propanamide | 0.089 |
| 22 | 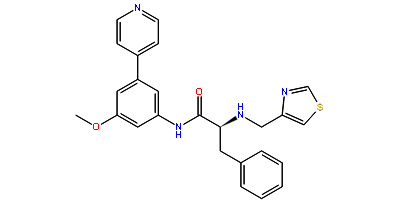 | C25H24N4O2S | (2S)-N-[3-methoxy-5-(pyridin-4-yl)phenyl]-3-phenyl-2-[(1,3-thiazol-4-ylmethyl)amino]propanamide | 0.053 |
| 23 | 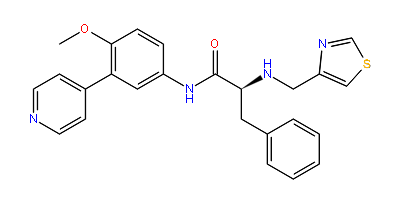 | C25H24N4O2S | (2S)-N-[4-methoxy-3-(pyridin-4-yl)phenyl]-3-phenyl-2-[(1,3-thiazol-4-ylmethyl)amino]propanamide | 0.24 |
| 24 | 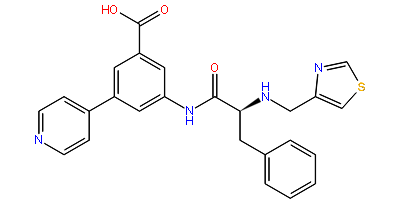 | C25H22N4O3S | 3-[(2S)-3-phenyl-2-[(1,3-thiazol-4-ylmethyl)amino]propanamido]-5-(pyridin-4-yl)benzoic acid | 0.11 |
| 25 | 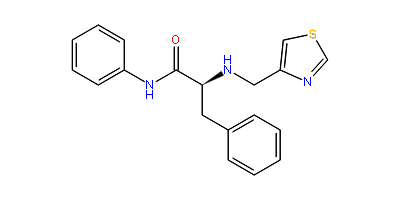 | C19H19N3OS | (2S)-N,3-diphenyl-2-[(1,3-thiazol-4-ylmethyl)amino]propanamide | 3.3 |
| 26 | 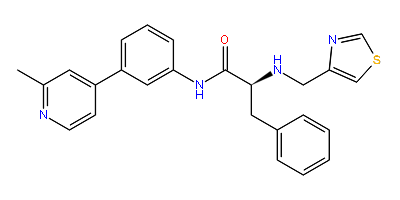 | C25H24N4OS | (2S)-N-[3-(2-methylpyridin-4-yl)phenyl]-3-phenyl-2-[(1,3-thiazol-4-ylmethyl)amino]propanamide | 0.052 |
| 27 | 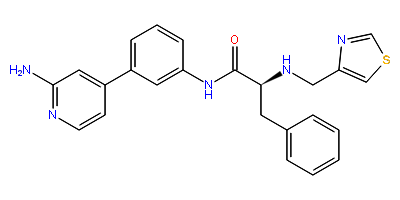 | C24H23N5OS | (2S)-N-[3-(2-aminopyridin-4-yl)phenyl]-3-phenyl-2-[(1,3-thiazol-4-ylmethyl)amino]propanamide | 0.21 |
| 28 | 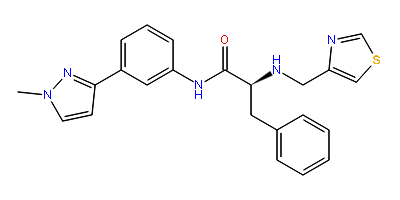 | C23H23N5OS | (2S)-N-[3-(1-methyl-1H-pyrazol-3-yl)phenyl]-3-phenyl-2-[(1,3-thiazol-4-ylmethyl)amino]propanamide | 1.9 |
| 29 | 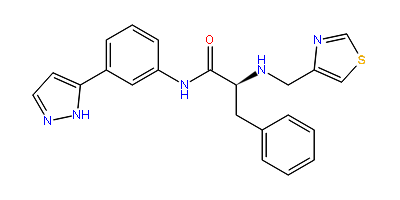 | C22H21N5OS | (2S)-3-phenyl-N-[3-(1H-pyrazol-5-yl)phenyl]-2-[(1,3-thiazol-4-ylmethyl)amino]propanamide | 0.067 |
| 30 | 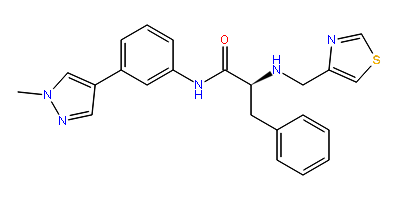 | C23H23N5OS | (2S)-N-[3-(1-methyl-1H-pyrazol-4-yl)phenyl]-3-phenyl-2-[(1,3-thiazol-4-ylmethyl)amino]propanamide | 0.18 |
| 31 | 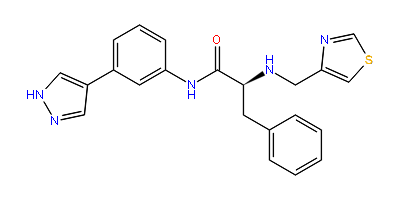 | C22H21N5OS | (2S)-3-phenyl-N-[3-(1H-pyrazol-4-yl)phenyl]-2-[(1,3-thiazol-4-ylmethyl)amino]propanamide | 0.18 |
| 32 | 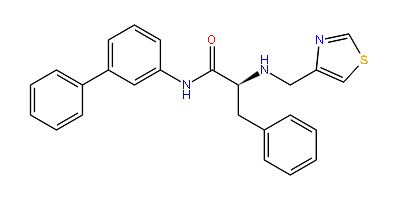 | C25H23N3OS | (2S)-3-phenyl-N-(3-phenylphenyl)-2-[(1,3-thiazol-4-ylmethyl)amino]propanamide | 0.64 |
| 33 | 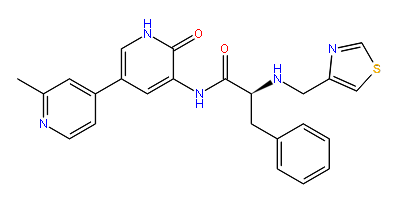 | C24H23N5O2S | (2S)-N-[5-(2-methylpyridin-4-yl)-2-oxo-1,2-dihydropyridin-3-yl]-3-phenyl-2-[(1,3-thiazol-4-ylmethyl)amino]propanamide | 0.067 |
| 34 | 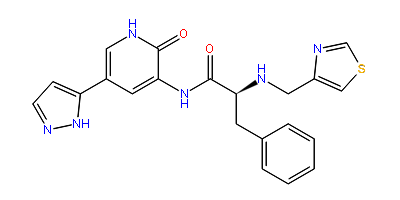 | C21H20N6O2S | (2S)-N-[2-oxo-5-(1H-pyrazol-5-yl)-1,2-dihydropyridin-3-yl]-3-phenyl-2-[(1,3-thiazol-4-ylmethyl)amino]propanamide | 0.20 |
| 35 | 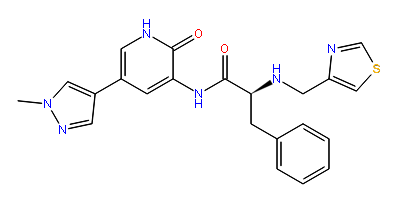 | C22H22N6O2S | (2S)-N-[5-(1-methyl-1H-pyrazol-4-yl)-2-oxo-1,2-dihydropyridin-3-yl]-3-phenyl-2-[(1,3-thiazol-4-ylmethyl)amino]propanamide | 0.23 |
| 36 | 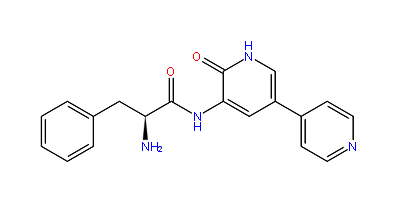 | C19H18N4O2 | (2S)-2-amino-N-[2-oxo-5-(pyridin-4-yl)-1,2-dihydropyridin-3-yl]-3-phenylpropanamide | 4.8 |
| 39 | 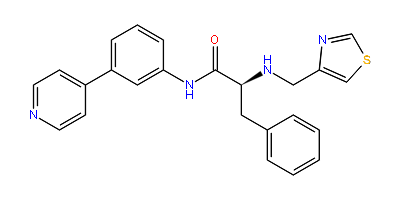 | C24H22N4OS | (2S)-3-phenyl-N-[3-(pyridin-4-yl)phenyl]-2-[(1,3-thiazol-4-ylmethyl)amino]propanamide | 0.067 |
| 40 | 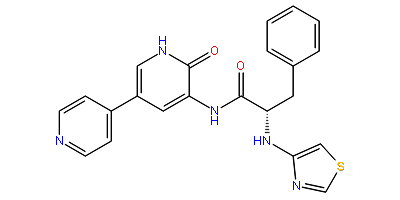 | C22H19N5O2S | (2S)-N-[2-oxo-5-(pyridin-4-yl)-1,2-dihydropyridin-3-yl]-3-phenyl-2-[(1,3-thiazol-4-yl)amino]propanamide | 0.93 |
| 41 | 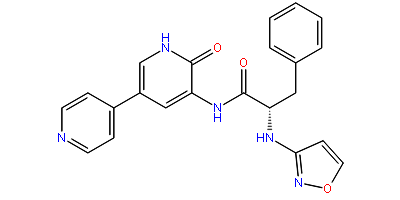 | C22H19N5O3 | (2S)-2-[(1,2-oxazol-3-yl)amino]-N-[2-oxo-5-(pyridin-4-yl)-1,2-dihydropyridin-3-yl]-3-phenylpropanamide | 2.2 |
| 42 | 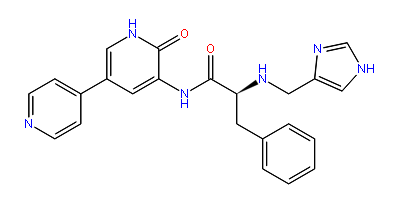 | C23H22N6O2 | (2S)-2-[(1H-imidazol-4-ylmethyl)amino]-N-[2-oxo-5-(pyridin-4-yl)-1,2-dihydropyridin-3-yl]-3-phenylpropanamide | 0.11 |
| 43 | 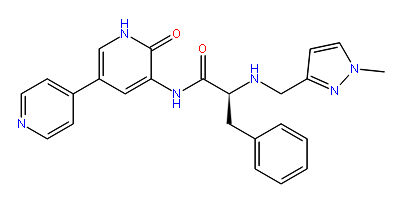 | C24H24N6O2 | (2S)-2-{[(1-methyl-1H-pyrazol-3-yl)methyl]amino}-N-[2-oxo-5-(pyridin-4-yl)-1,2-dihydropyridin-3-yl]-3-phenylpropanamide | 0.21 |
| 44 | 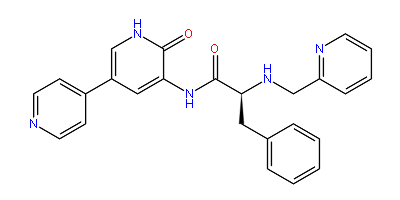 | C25H23N5O2 | (2S)-N-[2-oxo-5-(pyridin-4-yl)-1,2-dihydropyridin-3-yl]-3-phenyl-2-[(pyridin-2-ylmethyl)amino]propanamide | 0.095 |
| 45 | 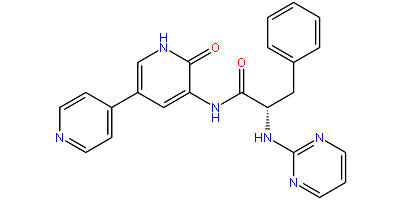 | C23H20N6O2 | (2S)-N-[2-oxo-5-(pyridin-4-yl)-1,2-dihydropyridin-3-yl]-3-phenyl-2-[(pyrimidin-2-yl)amino]propanamide | 0.44 |
| 46 | 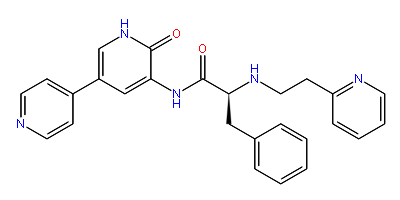 | C26H25N5O2 | (2S)-N-[2-oxo-5-(pyridin-4-yl)-1,2-dihydropyridin-3-yl]-3-phenyl-2-{[2-(pyridin-2-yl)ethyl]amino}propanamide | 1.32 |
| 48 | 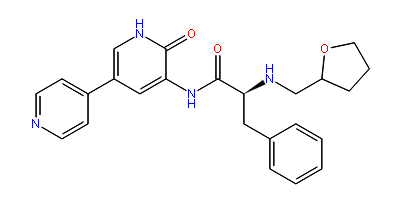 | C24H26N4O3 | (2S)-N-[2-oxo-5-(pyridin-4-yl)-1,2-dihydropyridin-3-yl]-2-[(oxolan-2-ylmethyl)amino]-3-phenylpropanamide | 0.39 |
| 49 | 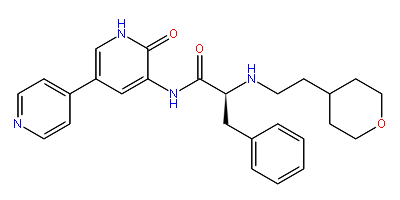 | C26H30N4O3 | (2S)-2-{[2-(oxan-4-yl)ethyl]amino}-N-[2-oxo-5-(pyridin-4-yl)-1,2-dihydropyridin-3-yl]-3-phenylpropanamide | 1.06 |
| 50 | 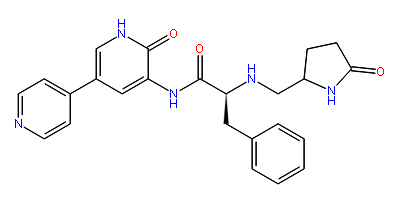 | C24H25N5O3 | (2S)-N-[2-oxo-5-(pyridin-4-yl)-1,2-dihydropyridin-3-yl]-2-{[(5-oxopyrrolidin-2-yl)methyl]amino}-3-phenylpropanamide | 2.23 |
| 51 | 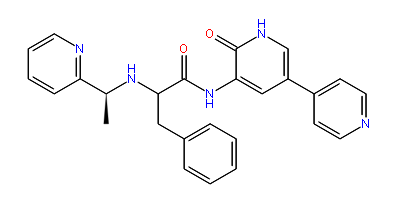 | C26H25N5O2 | N-[2-oxo-5-(pyridin-4-yl)-1,2-dihydropyridin-3-yl]-3-phenyl-2-{[(1S)-1-(pyridin-2-yl)ethyl]amino}propanamide | 0.22 |
| 52 | 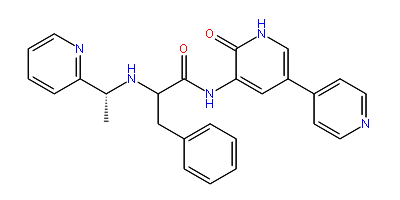 | C26H25N5O2 | N-[2-oxo-5-(pyridin-4-yl)-1,2-dihydropyridin-3-yl]-3-phenyl-2-{[(1R)-1-(pyridin-2-yl)ethyl]amino}propanamide | 6.0 |
| 53 | 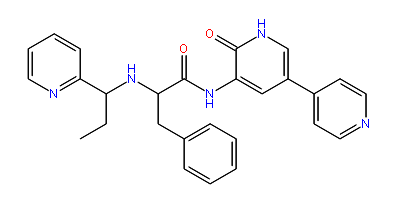 | C27H27N5O2 | N-[2-oxo-5-(pyridin-4-yl)-1,2-dihydropyridin-3-yl]-3-phenyl-2-{[1-(pyridin-2-yl)propyl]amino}propanamide | 0.73 |
| 54 | 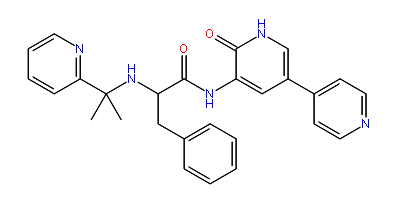 | C27H27N5O2 | N-[2-oxo-5-(pyridin-4-yl)-1,2-dihydropyridin-3-yl]-3-phenyl-2-{[2-(pyridin-2-yl)propan-2-yl]amino}propanamide | 9.45 |
| 55 | 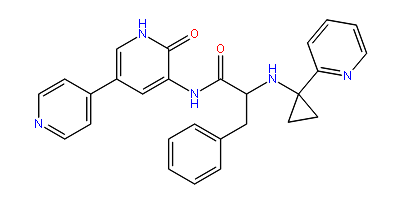 | C27H25N5O2 | N-[2-oxo-5-(pyridin-4-yl)-1,2-dihydropyridin-3-yl]-3-phenyl-2-{[1-(pyridin-2-yl)cyclopropyl]amino}propanamide | 0.054 |
| 56 | 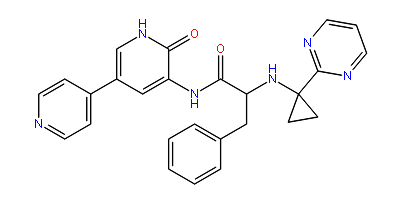 | C26H24N6O2 | N-[2-oxo-5-(pyridin-4-yl)-1,2-dihydropyridin-3-yl]-3-phenyl-2-{[1-(pyrimidin-2-yl)cyclopropyl]amino}propanamide | 0.099 |
| 57 | 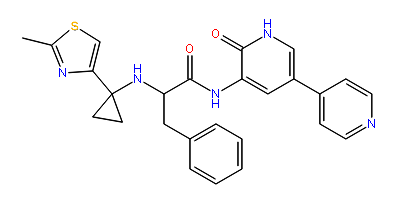 | C26H25N5O2S | 2-{[1-(2-methyl-1,3-thiazol-4-yl)cyclopropyl]amino}-N-[2-oxo-5-(pyridin-4-yl)-1,2-dihydropyridin-3-yl]-3-phenylpropanamide | 0.20 |
| 58 | 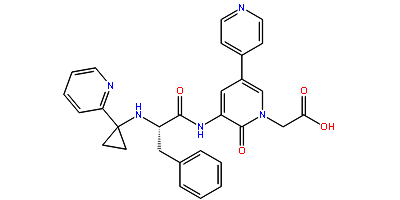 | C29H27N5O4 | 2-{2-oxo-3-[(2S)-3-phenyl-2-{[1-(pyridin-2-yl)cyclopropyl]amino}propanamido]-5-(pyridin-4-yl)-1,2-dihydropyridin-1-yl}acetic acid | 0.26 |
| 59 | 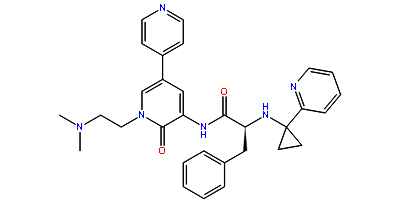 | C31H34N6O2 | (2S)-N-{1-[2-(dimethylamino)ethyl]-2-oxo-5-(pyridin-4-yl)-1,2-dihydropyridin-3-yl}-3-phenyl-2-{[1-(pyridin-2-yl)cyclopropyl]amino}propanamide | 0.28 |
| 60 | 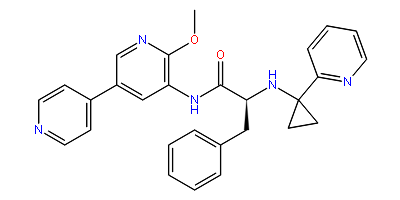 | C28H27N5O2 | (2S)-N-[2-methoxy-5-(pyridin-4-yl)pyridin-3-yl]-3-phenyl-2-{[1-(pyridin-2-yl)cyclopropyl]amino}propanamide | 0.35 |
| 61 | 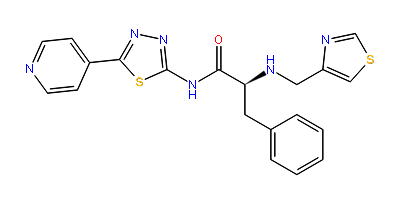 | C20H18N6OS2 | (2S)-3-phenyl-N-[5-(pyridin-4-yl)-1,3,4-thiadiazol-2-yl]-2-[(1,3-thiazol-4-ylmethyl)amino]propanamide | 0.086 |
| 62 | 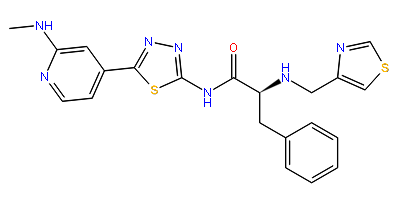 | C21H21N7OS2 | (2S)-N-{5-[2-(methylamino)pyridin-4-yl]-1,3,4-thiadiazol-2-yl}-3-phenyl-2-[(1,3-thiazol-4-ylmethyl)amino]propanamide | 0.036 |
| 63 | 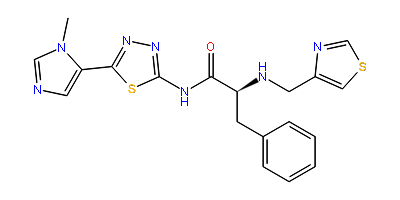 | C19H19N7OS2 | (2S)-N-[5-(1-methyl-1H-imidazol-5-yl)-1,3,4-thiadiazol-2-yl]-3-phenyl-2-[(1,3-thiazol-4-ylmethyl)amino]propanamide | 0.18 |
| 64 | 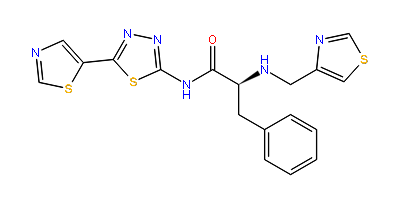 | C18H16N6OS3 | (2S)-3-phenyl-2-[(1,3-thiazol-4-ylmethyl)amino]-N-[5-(1,3-thiazol-5-yl)-1,3,4-thiadiazol-2-yl]propanamide | 0.39 |
| 66 | 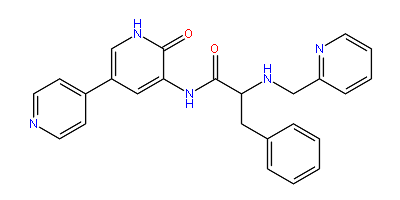 | C25H23N5O2 | N-[2-oxo-5-(pyridin-4-yl)-1,2-dihydropyridin-3-yl]-3-phenyl-2-[(pyridin-2-ylmethyl)amino]propanamide | 0.095 |

**Table S2-** Top 10 compounds are short listed from combined 3D Database of GPR142; where compound21 shown best docking affinity with penta ring structure that help to inhibit the Type 2 diabetes, where ADMET properties was calculated by ALOGPS2.1 program and basic feature were calculate using chemicalize program. Top 10 compounds are short listed from combined 3D Database of GPR142; where compound21 shown best docking affinity with penta ring structure that help to inhibit the Type 2 diabetes.

| Compound’s | Compound 2D | Molecular Formula | IUPAC Name | Docking Score (VSW) | Molecular Weight | LogP | LogS |
| --- | --- | --- | --- | --- | --- | --- | --- |
| Compound21 | 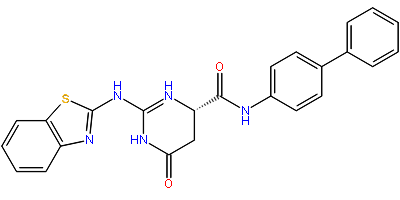 | C24H18N5O2S | (6S)-4-oxo-6-{[(4-phenylphenyl)amino]methaniumoyl}-2-[(1,3λ⁴-benzothiazol-2-ium-3a-ylium-3-id-2-yl)imino]-3,4,5,6-tetrahydro-1λ¹-pyrimidine-2,4-bis(ylium)-1-uide | -6.470 | 442.511g/mol | 3.00 | -5.88 |
| Compound22 | 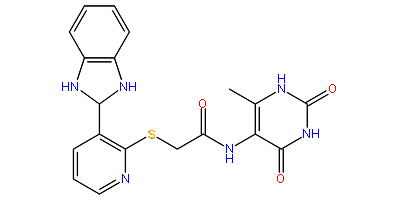 | C19H17N6O3S | 5-[(2-{[3-(1,3-benzodiazol-2-yl)-1,2-dihydropyridin-2-ylium-1-id-2-yl]sulfanyl}ethan-1-iumoyl)amino]-6-methyl-2,4-dioxo-1λ¹,3λ¹-pyrimidin-2-ylium-1,3-diuide | -7.060 | 409.442g/mol | -0.38 | -5.20 |
| Compound23 | 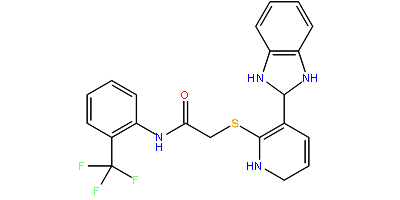 | C21H17F3N4OS | 2-{[3-(1,3-benzodiazol-2-yl)pyridin-2-yl]sulfanyl}-1-oxo-1-{[2-(trifluoromethyl)phenyl]amino}ethanium | -6.668 | 430.446g/mol | 0.65 | -6.76 |
| Compound24 | 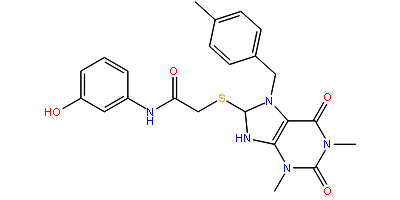 | C23H24N5O4S | 1,3-dimethyl-7-[(4-methylphenyl)methyl]-8-({2-[(3-oxidophenyl)amino]-2-oxoethan-2-ium-1-yl}sulfanyl)-2,3,6,7,8,9-hexahydro-1H-purine-2,6,8-tris(ylium)-2,6-bis(olate) | -6.826 | 466.532g/mol | 0.57 | -4.86 |
| Compound25 | 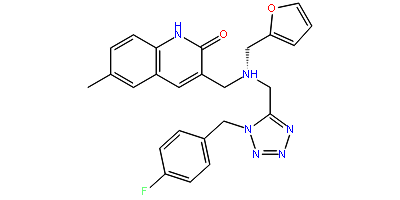 | C25H24FN6O2 | 3-{[({1-[(4-fluorophenyl)methyl]-1H-1,2,3,4-tetrazol-5-yl}methyl)(furan-2-ylmethyl)amino]methyl}-6-methyl-2-oxo-1,2-dihydroquinolin-2-ylium | -6.645 | 459.4949g/mol | 2.58 | -4.38 |
| Compound26 | 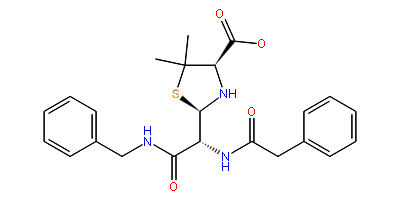 | C23H26N3O4S | (2R,4R)-2-[(1R)-2-(benzylamino)-2-oxo-1-[(2-phenylethan-1-iumoyl)amino]ethan-2-ium-1-yl]-4-[hydroxy(oxido)methyl]-5,5-dimethyl-1,3-thiazolidin-3-ide | -7.339 | 440.535g/mol | 2.34 | -4.90 |
| Compound27 | 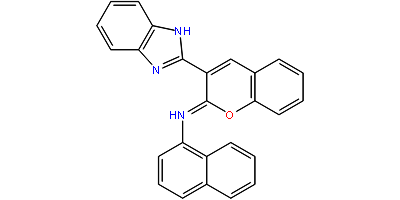 | C26H18N3O | 2-{2-[(naphthalen-1-yl)imino]-2H-chromen-2-ylium-3-yl}-1λ¹,3λ¹-benzodiazole-1,3-diuide | -7.161 | 388.441g/mol | 2.93 | -6.86 |
| Compound28 | 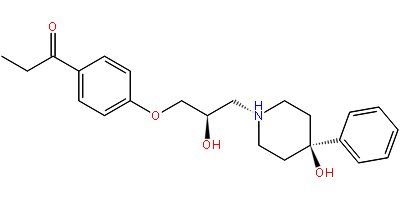 | C23H30NO4 | 1-{4-[(2R)-2-oxido-3-(4-oxido-4-phenylpiperidin-1-yl)propoxy]phenyl}-1-oxopropan-1-ium | -6.290 | 384.489g/mol | 2.15 | -5.17 |
| Compound29 | 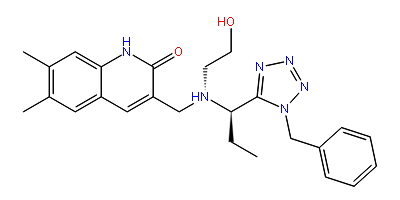 | C25H31N6O2 | 3-({[(1R)-1-(1-benzyl-1H-1,2,3,4-tetrazol-5-yl)propyl](2-oxidoethyl)amino}methyl)-6,7-dimethyl-2-oxo-1,2-dihydroquinolin-2-ylium | -6.742 | 447.552g/mol | 2.18 | -5.30 |
| Compound30 | 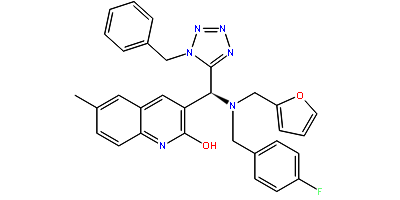 | C31H27FN6O2 | 3-[(S)-(1-benzyl-1H-1,2,3,4-tetrazol-5-yl)({[(4-fluorophenyl)methyl](furan-2-ylmethyl)azanidyl})methyl]-6-methyl-1λ¹-quinolin-2-ium-8a-ylium-1-uid-2-olate | -6.887 | 534.584g/mol | 4.72 | -4.04 |
| CompoundE1 | 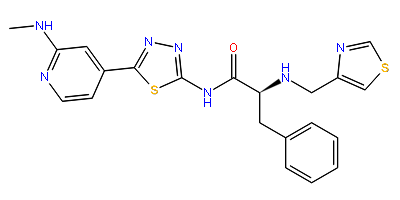 | C21H21N7OS2 | (2S)-N-{5-[2-(methylamino)pyridin-4-yl]-1,3,4-thiadiazol-2-yl}-3-phenyl-2-[(1,3-thiazol-4-ylmethyl)amino]propanamide | -4.860 | 450.56g/mol | 2.54 | -4.67 |

**Table S3-** Top 20 screened chemical compounds using virtual screening.

| Compounds | Compound 2D |  |
| --- | --- | --- |
| 01 | **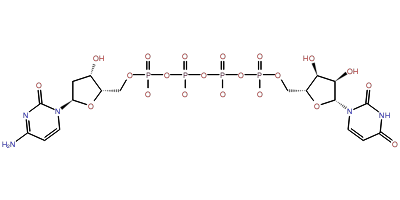** | C18H23N5O21P4 |
| 02 | **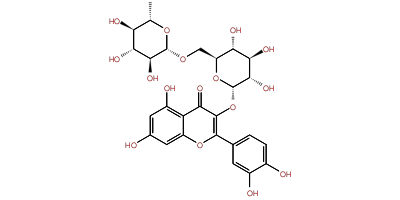** | C27H30O16 |
| 03 | **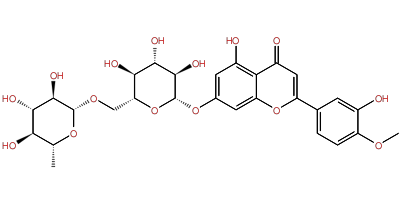** | C28H32O15 |
| 04 | **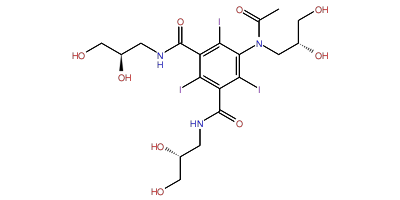** | C19H26I3N3O9 |
| 05 | **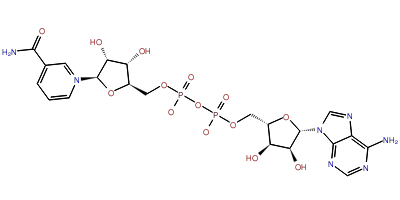** | C21H26N7O14P2 |
| 06 | **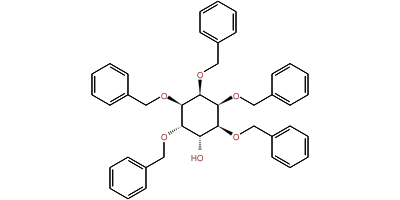** | C41H42O6 |
| 07 | **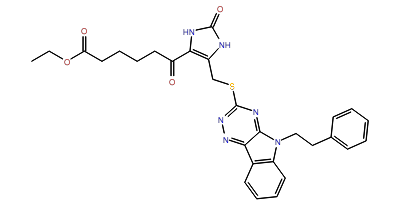** | C29H30N6O4S1 |
| 08 | **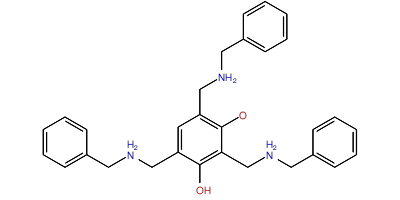** | C30H35N3O2 |
| 09 | **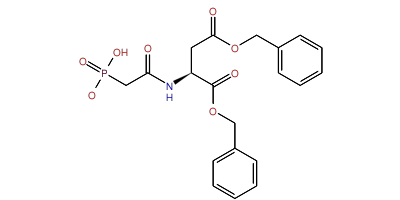** | C20H21N1O8P1 |
| 10 | **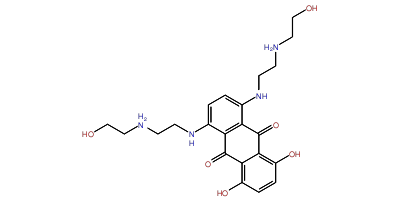** | C22H30N4O6 |
| 11 | **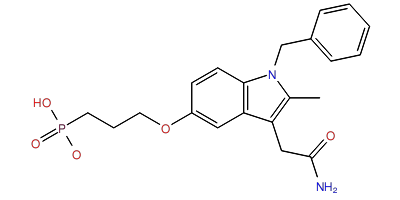** | C21H24N2O5P1 |
| 12 | **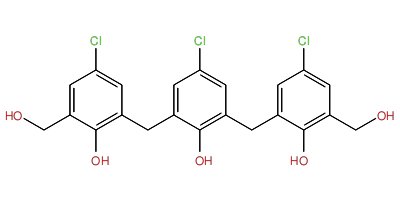** | C22H19Cl3O5 |
| 13 | **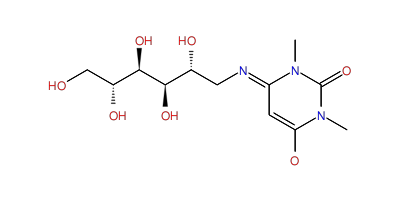** | C12H20N3O7 |
| 14 | **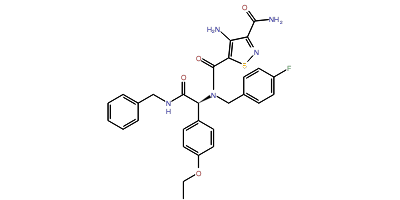** | C29H28F1N5O4S1 |
| 15 | **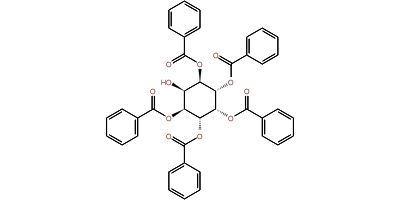** | C41H32O11 |
| 16 | **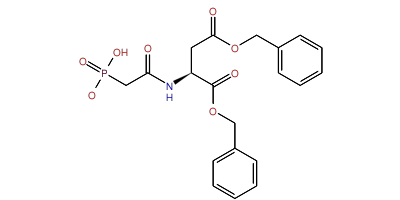** | C20H21N1O8P1 |
| 17 | **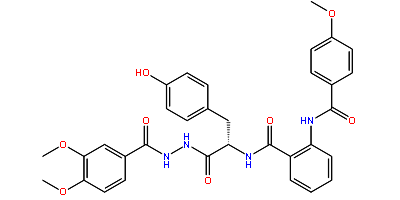** | C33H32N4O8 |
| 18 | **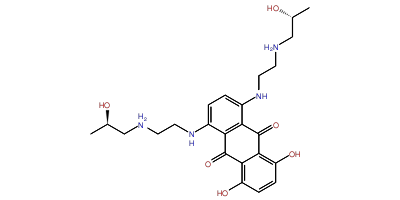** | C24H34N4O6 |
| 19 | **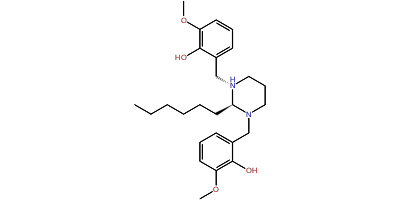** | C26H38N2O4 |
| 20 | **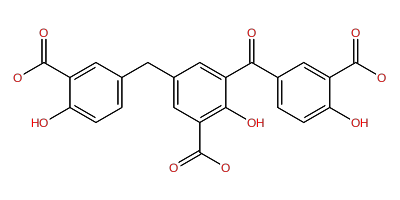** | C23H19NO10 |

**Figure S1:** Interaction fraction analysis of binding pocket residues with A) compound 2 and B) compound 21 during simulation. Residues order number are changed due to rearrangement of simulation system during building of MD system (Water model+ Membrane model+ Ions+ Protein Complexes).

**
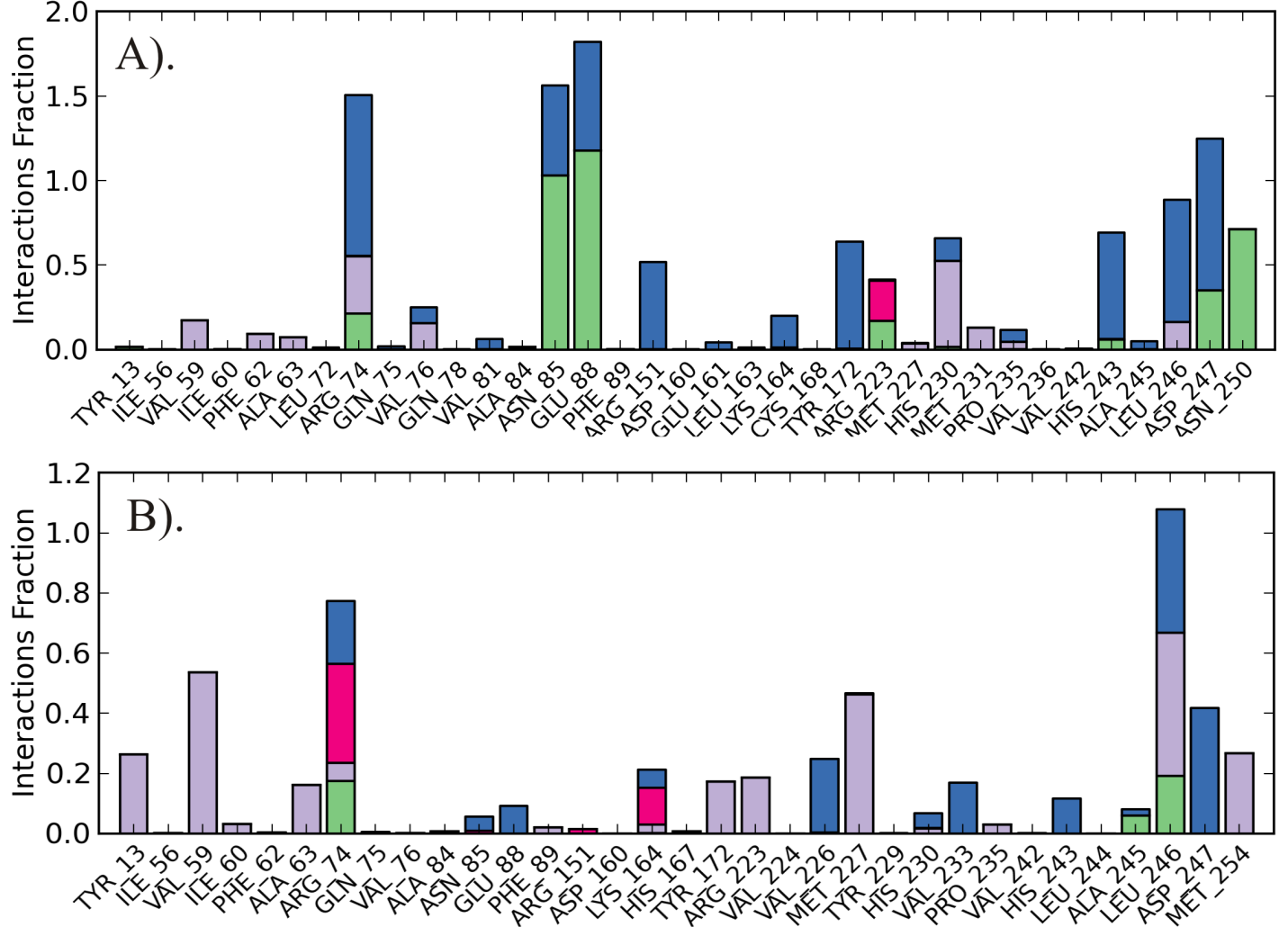
**
